# Cell‐Stress‐Free Percutaneous Bioelectrodes

**DOI:** 10.1002/adma.202509719

**Published:** 2025-09-09

**Authors:** Jungho Lee, Gaeun Yun, Juhyeong Jeon, Phuong Thao Le, Tae Sik Hwang, Jeongwoo Park, Jin‐Hyeok Baek, Seung Whan Kim, Hyoun Wook Lee, Kisang Kwon, Jihee Kim, Hoon Lim, Chulhong Kim, Sung‐Min Park, Geunbae Lim

**Affiliations:** ^1^ Department of Mechanical Engineering Pohang University of Science and Technology Pohang 37673 South Korea; ^2^ Department of Emergency Medicine College of Medicine Chungnam National University Daejeon 34134 South Korea; ^3^ Division of Interdisciplinary Bioscience and Bioengineering Pohang University of Science and Technology Pohang 37673 South Korea; ^4^ Department of Emergency Medicine Yongin Severance Hospital Yongin 16995 South Korea; ^5^ Department of Electrical Engineering Pohang University of Science and Technology Pohang 37673 South Korea; ^6^ Department of Biomedical Convergence Science and Technology Kyungpook National University Daegu 41566 Republic of Korea; ^7^ Department of Pathology Samsung Changwon Hospital Sungkyunkwan University School of Medicine Changwon 51353 South Korea; ^8^ Department of Clinical Laboratory Science Wonkwang Health Science University Iksan 54538 South Korea; ^9^ Department of Dermatology Yongin Severance Hospital Yongin 16995 South Korea; ^10^ Cutaneous Biology Research Institute Yonsei University College of Medicine Seoul 03722 South Korea; ^11^ Department of Emergency Medicine Soonchunhyang University Bucheon Hospital Bucheon 14584 South Korea; ^12^ Department of Convergence IT Engineering Pohang University of Science and Technology Pohang 37673 South Korea

**Keywords:** cell stress, microneedle, on‐skin bioelectrode, percutaneous, soft electronics

## Abstract

Wearable bioelectronics have advanced dramatically over the past decade, yet remain constrained by their superficial placement on the skin, which renders them vulnerable to environmental fluctuations and mechanical instability. Existing microneedle (MN) electrodes offer minimally invasive access to dermal tissue, but their rigid, bulky design—often 100 times larger and 10,000 times stiffer than dermal fibroblasts—induces pain, tissue damage, and chronic inflammation, limiting their long‐term applicability. Here, a cell‐stress‐free percutaneous bioelectrode is presented, comprising an ultrathin (<2 µm), soft MN (sMN) that dynamically softens via an effervescent structural transformation after insertion. The sMN exhibits near‐zero Poisson's ratio deformation, preserving cellular morphology and minimizing immune activation over multiple days of use in rats and humans. Synchrotron imaging and histological analysis reveal reduced tissue disruption, while electrophysiological measurements demonstrate stable signal‐to‐noise ratios under sweat, dehydration, and extended use. This architecture shifts the biosensing interface from the epidermis to the dermis, establishing a mechanically and electrically stable platform for environment‐independent signal acquisition. The findings establish dermal electronics as a next‐generation paradigm for long‐term, biocompatible wearable sensing.

## Graphical abstract

A structurally adaptive soft microneedle bioelectrode is developed with an effervescent sacrificial core that dissolves after insertion, leaving an ultrathin and highly compliant electrode integrated with skin. This design enables ultra‐flexible, cell‐stress‐free, stable, and high‐fidelity electrophysiological monitoring under dynamic conditions such as sweat and dehydration, surpassing conventional film and gel electrodes in both long‐term signal stability and mechanical compatibility.

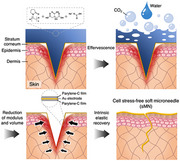

## 1 Introduction

The emergence of epidermal bioelectronics has catalyzed a golden era in wearable sensing technologies, ushering in devices capable of capturing physiological signals with remarkable sensitivity and comfort.^[^ ^1^, ^2^ ^]^ These skin‐conformal devices, often referred to as “epidermal sensors”, rely on ultrathin structures and soft materials to minimize the mechanical and electrical mismatch with the skin, thereby maximizing signal fidelity.^[^ ^3^, ^4^ ^]^ Their success lies in their ability to reduce the biosignal transmission path across the stratum corneum (SC) by adhering intimately to the skin's surface, aided by advanced material engineering such as stretchable electronics,^[^ ^5^, ^6^ ^]^ nanomeshes,^[^ ^7^, ^8^ ^]^ conductive hydrogels,^[^ ^9^, ^10^, ^11^, ^12^ ^]^ and liquid metals.^[^ ^13^ ^]^ These innovations have enabled unprecedented sensitivity in electromyography, electroencephalography, and biopotential recordings during motion or dynamic physiological conditions.^[^ ^14^, ^15^ ^]^

However, the paradigm of epidermal electronics has remained relatively static over the past decade. Despite improvements in mechanical compliance and signal amplification, these devices remain restricted to the skin surface, rendering them inherently vulnerable to external environmental fluctuations—such as sweat, skin hydration, and ambient noise—which degrade long‐term signal stability.^[^ ^16^, ^17^ ^]^ The use of surface electrodes also makes it difficult to isolate true physiological signals from environmental and motion‐related artifacts, a limitation that becomes especially pronounced in wearable systems designed for continuous monitoring. Thus, while epidermal electronics have excelled in short‐term or controlled settings, their utility in real‐world, long‐term monitoring remains constrained by their superficial location and exposure to variable skin conditions.

In contrast, fully implantable electrodes have demonstrated exceptional signal quality due to their placement deep within the body, where they are naturally shielded from external perturbations. These systems, though lacking in wearability, offer direct interfacing with target tissues and have been critical in applications such as brain–machine interfaces,^[^ ^18^ ^]^ cardiac monitoring,^[^ ^10^ ^]^ and closed‐loop neural stimulation.^[^ ^19^ ^]^ The success of these devices has been amplified by advances in wireless power delivery,^[^ ^20^ ^]^ stretchable circuits,^[^ ^5^, ^6^ ^]^ and biocompatible encapsulation.^[^ ^21^, ^22^, ^23^ ^]^ Yet, their inherent invasiveness and surgical requirements limit their practicality, often confining their use to high‐risk patients and clinical environments. Moreover, their long‐term safety and immune compatibility remain subjects of ongoing concern.

This technological divide has motivated the development of semi‐implantable devices that combine the user‐friendly interface of epidermal sensors with the signal fidelity of implantable systems.^[^ ^24^ ^]^ Among these, microneedle (MN) electrodes have gained significant attention.^[^ ^25^, ^26^, ^27^, ^28^, ^29^ ^]^ Their minimally invasive nature allows for access to the dermis without surgical procedures,^[^ ^30^ ^]^ and their microscale dimensions enable MEMS‐based precision engineering, which even enables stretchable MN arrays.^[^ ^27^ ^]^ By penetrating the SC, MNs bypass the high‐impedance barrier of the outer skin, directly interfacing with lower‐resistance dermal tissue and offering a more stable electrical pathway.^[^ ^31^, ^32^ ^]^ However, most MNs are designed to be mechanically robust in order to achieve effective skin penetration. Ironically, this very robustness—often involving structures 10 times larger and 100 times stiffer than skin cells—leads to cumulative tissue damage, pain, and inflammation during prolonged use.^[^ ^33^, ^34^, ^35^, ^36^, ^37^ ^]^ Thus, while MNs present a rational compromise between implantable and epidermal systems, their long‐term utility remains unproven due to the mechanical mismatch at the tissue interface.

Efforts to improve MN biocompatibility have focused on softening the supporting substrate^[^ ^25^, ^26^, ^27^, ^28^, ^29^ ^]^or minimizing insertion depth,^[^ ^38^ ^]^ but these approaches fall short of addressing the fundamental problem: the persistent presence of bulky, rigid materials embedded in soft biological tissue. Approaches that succeed in soft‐tissue environments—such as ultra‐flexible brain electrodes^[^ ^39^ ^]^ or dissolvable vascular implants^[^ ^40^ ^]^—have yet to demonstrate analogous success in skin applications. Unlike the epidermis, which is highly susceptible to hydration fluctuations and external disturbances, the dermal layer provides a relatively stable electrical and mechanical environment.^[^ ^41^ ^]^ This makes it an attractive target for semi‐implantable biointerfaces, provided they can maintain mechanical compatibility and immune tolerance over long‐term use. However, realizing such dermal‐level integration requires devices that can seamlessly adapt to the dynamic mechanical deformation of skin while avoiding chronic immune activation. To highlight the design trade‐offs of previous MN systems and the unique integration strategy of our approach, we provide a comparative summary of key electrode characteristics in Table S1 (Supporting Information).

Here, we introduce a structurally adaptive, cell‐stress‐free percutaneous soft MN (sMN) bioelectrode that overcomes these limitations. Our design integrates a mechanically rigid, effervescent scaffold that dissolves upon insertion, leaving behind an ultrathin, highly compliant electrode. This reconfiguration reduces both the geometric and mechanical footprint of the electrode, yielding a structure more than ten times thinner and ten times softer than dermal fibroblasts. The sMN conforms to the skin's dynamic curvature, avoids accumulation of mechanical stress at the tissue interface, and eliminates residual fragments that could otherwise trigger immune responses. Importantly, the electrode's soft mechanics result in near‐zero Poisson's ratio deformation, enabling mechanical adaptability without lateral strain on surrounding cells.

We validate this design using a suite of in vivo and ex vivo experiments. High‐resolution synchrotron micro‐CT imaging reveals volumetric deformation and stress dissipation throughout the unique hollow and thin MN structure. Histological analysis quantifies reductions in inflammatory response and micropore persistence compared to conventional bulky MNs (bMNs) and flexible MNs (fMNs). The fMN is 10 times thicker than the sMN and exhibits high mechanical compliance but lacks adequate softness and flexibility. Functionally, the sMN achieves noise‐independent, long‐term signal acquisition of electromyographic activity under conditions of sweating, dehydration, and mechanical motion—outperforming gel and film‐based electrodes in both signal fidelity and duration of stable operation.

By uniting the biophysical advantages of subdermal sensing with the practical benefits of wearability, our work redefines the interface between skin and electronics. This percutaneous architecture represents a step beyond epidermal electronics toward a new class of dermal electronics—systems that maintain signal integrity across time, environment, and user variability without compromising comfort or biocompatibility.

## 2 Results

### 2.1 Effervescent Transformation Enables Ultra‐Soft MN Electrodes

**Figure 1:**
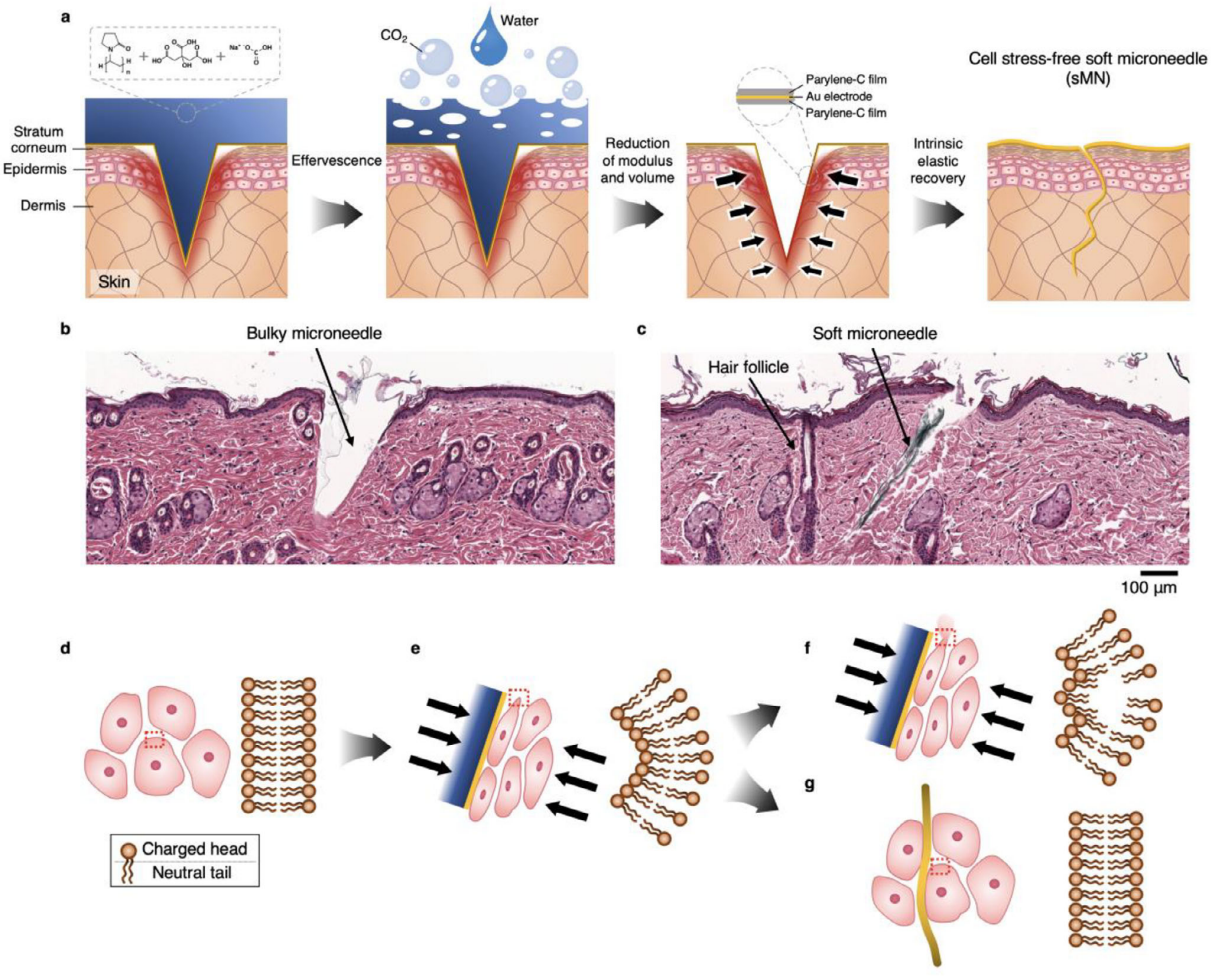
Mechanism of action and tissue compatibility of the cell‐stress‐free percutaneous bioelectrode. a), Schematic illustration of the effervescent transformation of the soft microneedle (sMN) bioelectrode. Before effervescence, the sMN—composed of polyvinylpyrrolidone (PVP), citric acid, and sodium bicarbonate—penetrates the skin while exerting transient mechanical stress, as indicated by localized red shading. When a small amount of water is applied, an effervescent reaction occurs, producing CO_2_ bubbles and dissolving the sacrificial matrix. As the reactive components vanish, the surrounding skin partially closes, leaving only the ultrathin parylene–gold–parylene film electrode in place. The process completes with the skin pores fully closed and the flexible sMN conformally integrated with the tissue, minimizing residual stress. b,c), Hematoxylin and eosin (H&E)–stained cross‐sections of Sprague–Dawley (SD) skin with bMN (b) and sMN (c) insertions. The bMN causes noticeable tissue deformation, whereas the sMN conforms with minimal disruption. A nearby hair follicle of similar size and shape is visible in the section. d–g), Schematic of cell–microneedle interactions. Cells initially maintain a stable shape with intact phospholipid bilayers (d). A rigid MN compresses cells, deforming and destabilizing their membranes (e), which can lead to bilayer disruption and damage under sustained stress (f). When the MN softens rapidly, cells recover their shape and membrane integrity, allowing the sMN to settle between cells without further damage (g).

To understand how the sMN achieves mechanical biocompatibility, we first examined its transition mechanism and cellular interface following implantation. **Figure** **1**a illustrates the working principle of the sMN, which initially resembles a conventional bMN that is mechanically rigid and significantly larger than surrounding skin cells. Figures S1 and S2 (Supporting Information) show the fabrication process for the sacrificial MN patch. Upon exposure to water, the effervescent sacrificial MN patch dissolves with vigorous CO_2_ bubble formation, leaving a pre‐deposited ultra‐thin film electrode partially implanted within the skin (Figure S3 and Video S1, Supporting Information; fabrication details are provided in Methods). This phase transition is enabled by a carefully engineered effervescent sacrificial polymer composite that balances rapid dissolution kinetics with initial mechanical stiffness. The sacrificial core completes dissolution within 5 min under physiological conditions (25 °C, pH ≈7.5), producing consistent CO_2_ foaming without visible residue or tissue damage, as confirmed by fluorescence‐traced residue clearance (Figure S4, Supporting Information). The buffering action of sodium citrate, a reaction byproduct, stabilizes pH throughout the process, maintaining a near‐neutral environment and preventing acid–base or osmotic stress. Furthermore, in vitro cytotoxicity was assessed by exposing fibroblasts to a 5% (v/v) eluate for 24 and 48 h, revealing no significant decrease in viability (Figure S5, Supporting Information), thereby confirming the excellent biocompatibility of the effervescent components. The transformation yields an ultrathin (approximately ten times thinner than a dermal fibroblast) and compliant thin‐film electrode with an effective modulus of ≈35 to 28 MPa—comparable to soft tissue, depending on its hydration degree—allowing it to conform intimately to the skin.

The mechanical benefit of this transition is amplified by the intrinsic elasticity of skin tissue. Following effervescence, the tissue's natural recoil closes the micropores left by the MN insertion,^[^ ^42^, ^43^ ^]^ effectively encapsulating the soft electrode within the dermis and eliminating fluid gaps that would otherwise impede signal transmission.^[^ ^29^, ^44^ ^]^ Histological sections of Sprague–Dawley (SD) rat skin confirm these effects: bMNs leave behind visible disruption and cavity formation (Figure 1b), whereas sMNs induce minimal structural disturbance and exhibit a tapered morphology resembling that of hair follicles, allowing for intimate integration with surrounding tissue (Figure 1c). The mechanical compliance of the sMN makes it nearly indistinguishable from the surrounding tissue in both gross morphology and microscopic architecture.

At the cellular scale, the interface becomes particularly important. The phospholipid bilayer of the cell membrane, shown in Figure 1d, is held together by lateral electrostatic forces that maintain its curvature and fluidity.^[^ ^45^ ^]^ Excessive pressure from rigid devices like bMNs can disrupt this electrostatic balance (Figure 1e), leading to microstructural deformation and potentially initiating immune cascades (Figure 1f).^[^ ^46^, ^47^ ^]^ In contrast, the sMN conforms to the native curvature of adjacent cells (Figure 1g), distributing pressure over a wider contact area and avoiding membrane rupture. This biophysical harmony forms the foundational rationale for the sMN's designation as “cell‐stress‐free.”

### 2.2 Mechanical Flexibility and Electrical Durability of Ultrathin sMN

**Figure 2:**
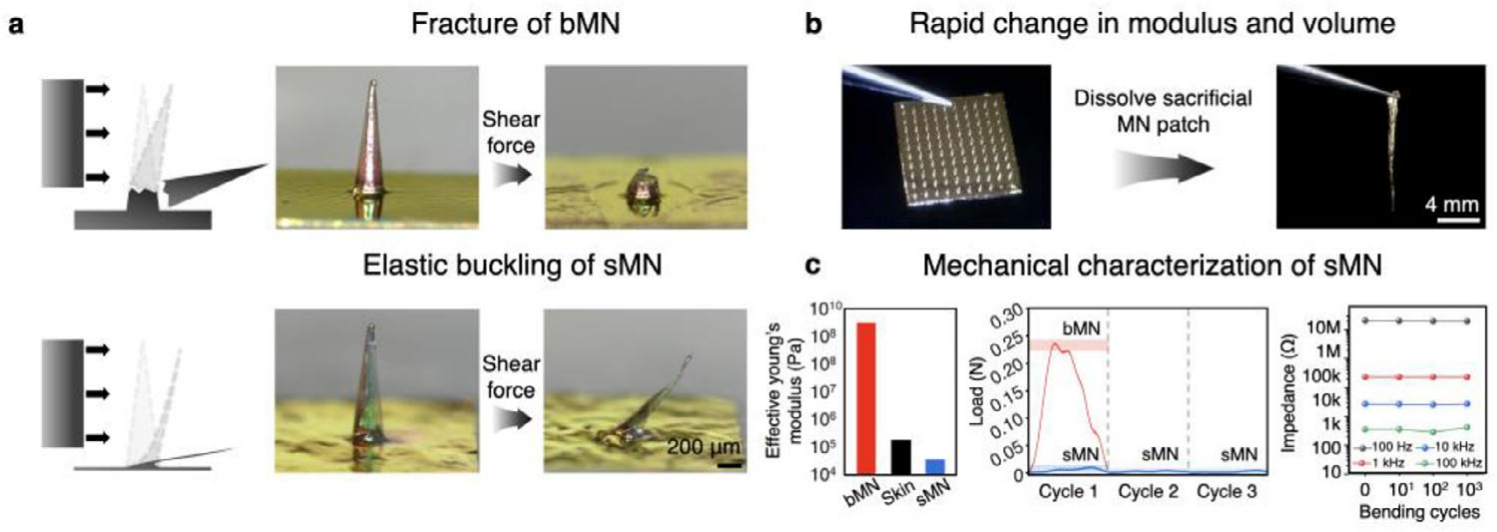
Mechanical transformation and durability of the sMN bioelectrode. a), Schematic and optical images showing that bMN fractures under shear force (top), whereas post‐effervescence (softened) sMN undergoes elastic buckling without damage (bottom). b), Photographs before and after effervescence, illustrating the transition from a rigid patch to an ultrathin, flexible sMN. c), Comparison of the effective Young's modulus of bMN, skin tissue, and softened sMN, showing that the sMN achieves a significantly lower modulus (34.5–27.8 MPa) than bMN (3.6–3.9 GPa), enabling superior mechanical compliance (Left). Load–displacement curves under three shear cycles, where bMN fails on the first cycle while sMN remains stable (Middle). Impedance of sMN at 100 Hz–100 kHz after 0–1000 bending cycles, showing negligible electrical change (Right).

To investigate how mechanical stress is minimized in the embedded state of sMNs, we analyzed the structural response of both bMN and sMN electrodes to shear force (Figure S6, Supporting Information). As shown in **Figure** **2**a, the bMN, composed of a rigid polymer core, fractures under lateral shear, confirming its mechanical fragility and incompatibility with repeated skin movement. In contrast, the sMN exhibits higher mechanical compliance than bMNs, undergoing elastic buckling rather than structural failure due to its ultrathin architecture and intrinsic flexibility (Figure S7, Supporting Information). This deformation behavior enables the sMN to absorb and redistribute applied forces rather than concentrate stress at the insertion site.

This transformation results from the dissolution of the effervescent sacrificial core, which reduces the electrode's mass by ≈99.7% and leaves behind an ultrathin (1.1 µm) parylene‐C film (Figure 2b; Figure S8, Supporting Information). All measurements were conducted on the sMN after effervescent core removal, ensuring that the results reflect the mechanical and electrical properties of the final soft microneedle structure. Although parylene‐C itself is a stiff polymer (bulk Young's modulus ≈2–3 GPa), the effervescently thinned hollow architecture of the sMN exhibits a deformation‐dependent effective Young's modulus ranging from ≈35 to ≈28 MPa (Figure 2c), which is lower than or comparable to the modulus of (epi)dermal tissue (200 kPa–1 MPa) under low‐strain conditions, as measured under physiologically relevant compression (Figure S9, Supporting Information). This dramatic reduction in structural stiffness allows the sMN to conform seamlessly to soft biological tissues. In comparison, the bMN retains a bulk modulus of ≈3.6 to 3.9 GPa, which is more than two orders of magnitude stiffer than skin tissue (typically 200 kPa–1 MPa, depending on hydration and anatomical site).^[^ ^48^ ^]^

Quantitative mechanical characterization further highlights the sMN's advantages. As shown in Figure 2c (left), the bMN's high modulus contrasts sharply with the sMN, whose mechanical compliance exceeds that of native skin. In the repeated shear displacement test (Figure 2c, middle), the bMN fractured abruptly upon the first loading, indicating poor tolerance to lateral stress and precluding further fatigue testing. In contrast, the sMN maintained structural integrity across three repeated shear cycles. A modest reduction in peak force was observed after the first cycle, which we attribute to hysteresis‐induced softening and inner core stabilization. After this initial adaptation, the sMN exhibited a consistent mechanical response without further degradation, suggesting a fatigue‐resilient and compliant architecture optimized for long‐term percutaneous integration.

We performed dynamic electrochemical durability tests to evaluate electrode stability under repetitive mechanical strain. Cyclic voltammetry (CV) profiles measured before and after 100 cycles of 180° bending showed minor variations, which are attributed to slight geometric changes in the soft MN during deformation; however, the overall redox behavior and double‐layer capacitance remained stable and reproducible (Figure S10, Supporting Information). Impedance spectra at 100 Hz, 1 kHz, 10 kHz, and 100 kHz were further measured after 0, 10, 100, and 1000 bending cycles (Figure 2c, right; Figure S11; Video S2, Supporting Information), showing less than ±5% variation at the key low‐frequency ranges (100 Hz and 1 kHz) critical for biosignal acquisition. These results confirm that the sMN retains both mechanical compliance and electrochemical performance under prolonged dynamic stress, ensuring robust and reliable operation for long‐term epidermal or percutaneous biosignal monitoring.

### 2.3 Multiaxial Adaptability and Stress‐Dissipating Behavior in Curved Skin

**Figure 3:**
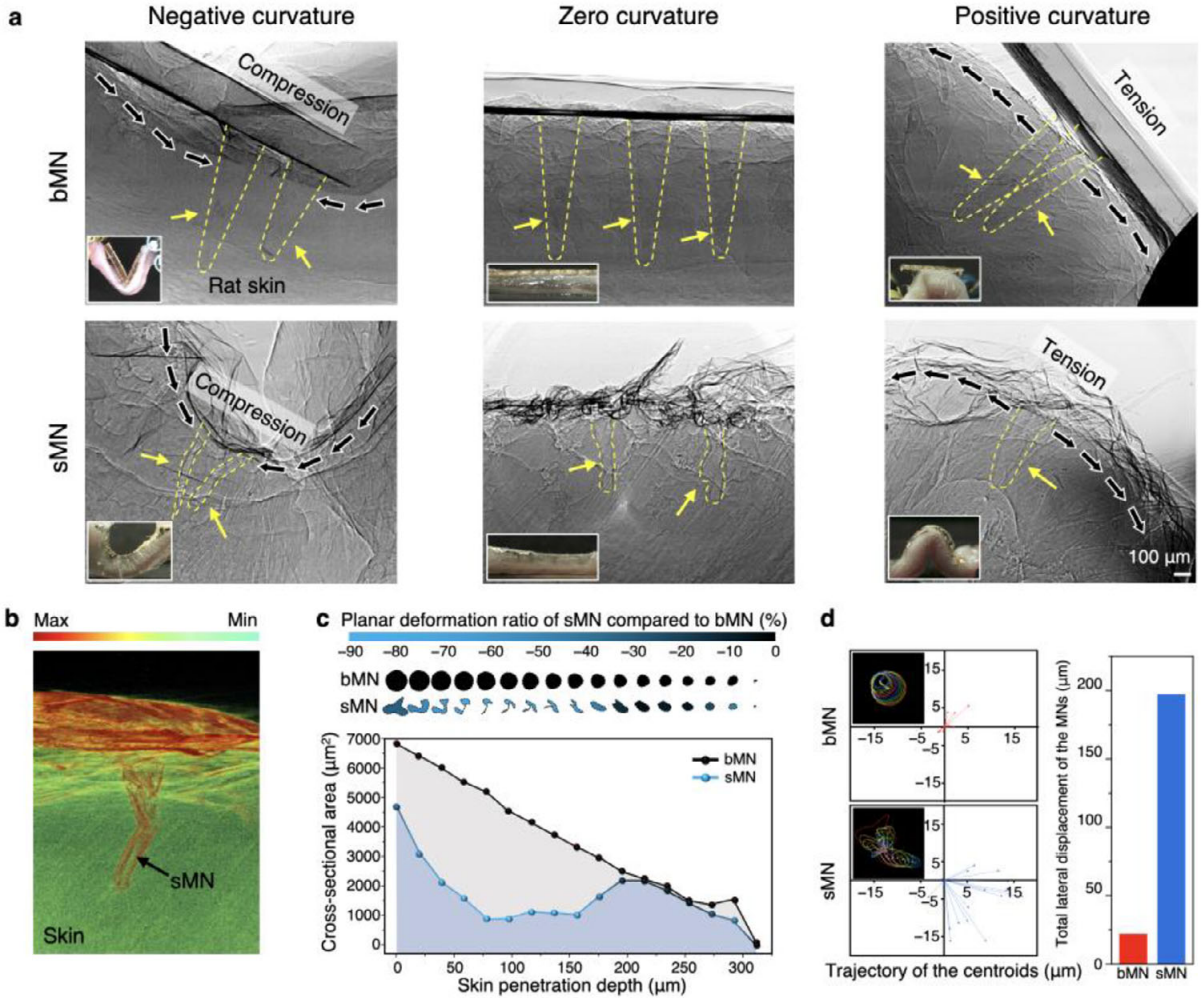
Mechanical adaptability of and deformation behavior of bMN and sMN. a), Synchrotron X‐ray images of bMN (top) and sMN (bottom) under negative, zero, and positive curvature of rat skin, with insets showing patch conformality. bMN fractures or detaches under compression or tension, while sMN maintains conformal contact, compressing or spreading laterally as needed. b), 3D synchrotron micro‐CT rendering of sMN with intensity‐based colormap, illustrating flexible deformation inside tissue. c), Cross‐sectional area analysis along insertion depth, where bMN shows a steady taper, whereas sMN exhibits variable expansion and contraction (maximum planar deformation ≈46%). d, Trajectory of the centroids along the MN axis, showing lateral displacement of sMN and bMN (left). Quantified total lateral displacement, highlighting ≈9 times higher adaptability of sMN compared to bMN (right).

To examine how the sMN accommodates mechanical deformation after insertion into the skin tissue of the SD rat, we performed *ex vivo* imaging while bending the curved skin surface. Using synchrotron X‐ray imaging (Figure S12, Supporting Information), we observed that the sMN, comprising an ultrathin MN tip and compliant substrate, accommodates various curvature states—compression (negative curvature), neutral (flat), and tension (positive curvature)—through multidirectional bending and shape adaptation (**Figure** **3**a). Critically, the MN tips themselves undergo volumetric deformation inside the dermal tissue, dynamically reducing their internal volume and conforming to the surrounding cellular environment. This unique volume‐compliance mechanism allows the sMN to minimize mechanical stress accumulation at the tissue–device interface, in contrast to conventional rigid bMNs that resist deformation and cause localized microtrauma.

These curvature‐adaptive properties of the sMN were further validated through high‐resolution 3D micro‐CT reconstruction (Figure 3b; Figures S13 and S14, and Video S3, Supporting Information),^[^ ^49^ ^]^ which enabled in situ visualization of volumetric deformation. Unlike the bMN, which retains its original shape and volume post‐insertion, the sMN undergoes structural adaptations—such as twisting, buckling, and localized volume reduction—in alignment with external mechanical loading. In contrast, while bulk parylene, which has a Poisson's ratio of ≈0.4, the hollow, thin‐walled architecture of the sMN suppresses lateral expansion under distributed tissue pressure, effectively exhibiting structure‐induced low lateral strain. This behavior, which mimics a near‐zero Poisson's response, arises from buckling and shell‐like collapse rather than material properties, as confirmed by Micro‐CT imaging under uniaxial and quasi‐isotropic compression (Figure S15, Supporting Information).

Cross‐sectional analysis (Figure 3c) revealed that these deformations are not uniformly distributed, but are instead concentrated at a depth of 60–80 µm, corresponding to regions of maximal shear and compressive stress within the skin. Quantification of cross‐sectional area along the needle depth showed a maximum volume reduction of 45.93% for the sMN, compared to a negligible change in the bMN. These findings support the hypothesis that the sMN functions as a stress‐dissipating layer, absorbing localized mechanical energy and preventing its propagation to adjacent tissues. Notably, this deformation occurred without fracture or delamination, consistent with the sMN's elastic buckling behavior and mechanical resilience demonstrated in Figure 2.

To capture more physiologically relevant deformations, we extended these analyses beyond uniaxial positive/negative curvature tests to include biaxial shear and in‐plane stretching/compression experiments (Figure S16, Supporting Information). After 100 cycles of biaxial strain (5–10% in‐plane stretching and compression), the sMN maintained its structural integrity without visible damage or delamination, as confirmed by Micro‐CT (Figure S17, Supporting Information).

To further assess dynamic deformation, we traced the centroids of cross‐sectional layers along the MN axis (Figure 3d). The sMN underwent substantial lateral displacement, totaling 196.96 µm—almost 9 times greater than the bMN. This suggests that the sMN can accommodate internal tissue motion, such as skin stretch or compression during physical activity, without causing localized stress accumulation.

Electrochemical performance was also preserved under these conditions. Broadband impedance measurements (100 Hz–100 kHz) before and after the repeated biaxial loading showed <5% variation, with stable low‐frequency impedance at 1 kHz—critical for biosignal acquisition (Figure S18, Supporting Information). This result indicates that both mechanical compliance and electrode functionality of the sMN are maintained under realistic multidirectional deformations.

Furthermore, we performed finite element simulations to quantify the stress–strain distributions within the tissue upon external compression after MN insertion. Volumetric strain maps revealed that bMNs transmit concentrated stress directly to the surrounding dermis, particularly near the MN‐tissue interface, whereas sMNs exhibit internal space collapse that mitigates mechanical strain transmission (Figure S19, Supporting Information).

Together, these results demonstrate that the sMN not only integrates physically into the dermal layer, but also behaves mechanically as an adaptive interface that buffers against in vivo mechanical perturbation. This unique combination of volume compliance, multiaxial flexibility, and structural resilience supports its function as a next‐generation stress‐free percutaneous electrode.

### 2.4 Quantitative Assessment of User Discomfort and Skin Compatibility

**Figure 4:**
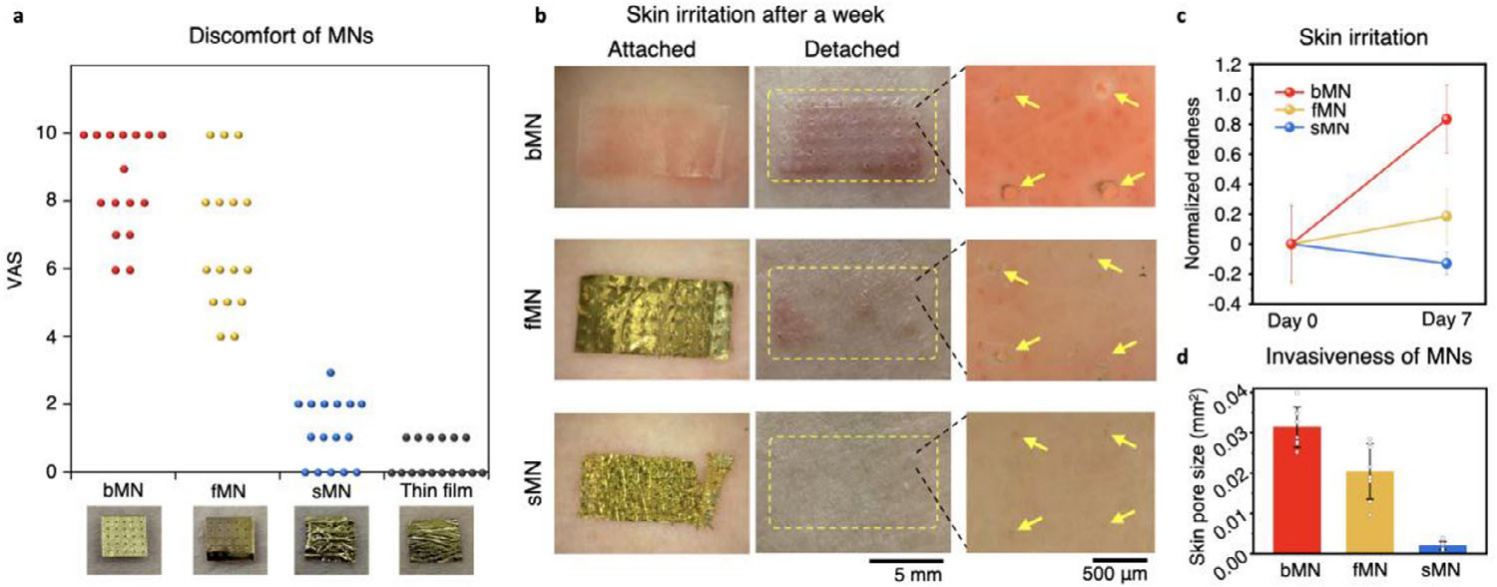
Human‐subject evaluation of discomfort and skin irritation after electrode use. a), Visual analogue scale (VAS) scores from participants reporting discomfort associated with four types of electrodes: bMN, fMN, sMN, and thin‐film. b), Representative images of the skin before and immediately after detachment of the device, taken one week after initial application. Yellow dashed boxes indicate device location; yellow arrows mark residual micropores. c), Quantification of skin redness over 7 days, normalized to the baseline level measured at day 0, where all groups (bMN, fMN, and sMN) exhibited comparable redness. d) Micro pore closure rates were analyzed using one‐way ANOVA. Results are presented as mean ± s.d., with *n* = 8 per group, which shows the statistical significance between groups (Thin film, sMN, fMN, bMN) using significance markers (^*^ *p* < 0.05, ^**^ *p* < 0.01, ^***^ *p* < 0.001, see Tables S2 and S3, Supporting Information).

To evaluate user experience and dermatological compatibility of the sMN electrode, we conducted a human‐subject study focused on perceived discomfort and skin irritation. In a blind test using the visual analogue scale (VAS), participants were asked to report the level of discomfort while wearing each of four electrode types: bMN, fMN, sMN, and conventional thin‐film electrodes (**Figure** **4**a; Figure S20, Supporting Information). The bMN received the highest discomfort scores (VAS 6–10), followed by fMN (VAS 4–10). In contrast, both sMN and thin‐film electrodes consistently scored below VAS 2, indicating near‐zero discomfort during application.

We further assessed skin response over time following continuous electrode wear. Representative images of skin before and after detachment revealed that bMNs produced visible erythema and micropore traces after one week of wear (Figure 4b, top). The fMN group exhibited milder irritation (Figure 4b, middle), while the sMN left no visible redness or pores (Figure 4b, bottom). These qualitative results were supported by quantitative measurement of skin redness normalized to baseline, showing a sharp increase in bMN‐applied skin, a moderate increase in fMN‐applied skin, and minimal change in sMN‐applied skin over the 7‐day period (Figure 4c). The moderate response of fMN is likely attributable to its intermediate stiffness—being harder than sMN but softer than bMN (Figure S21, Supporting Information). Notably, erythema in the bMN and fMN groups persisted even after device removal (Figures S22 and S23, Supporting Information), indicating that structural characteristics such as rigidity and bulkiness correlate with prolonged irritation.

These differences can be attributed to the physical footprint left on the skin by each electrode. While bMNs generated large and irregular micropores, fMNs left moderately sized punctures. In contrast, the sMN's ultrathin and soft structure allowed the skin to elastically recover and close micropores rapidly after device removal (Figure 4d). The absence of surface debris or epithelial scarring in the sMN group is attributed to the minimal insertion volume and soft‐tissue compliance of the parylene‐C film (Video S4, Supporting Information). Moreover, the sMN's mechanical transition post‐insertion eliminates rigid remnants that would otherwise remain inside the skin, contributing to its superior dermatological compatibility.

To further corroborate these findings in a clinically relevant context, we performed a long‐term application study on human skin under dermatological supervision. Over 19 consecutive days, the sMN site exhibited only mild and transient erythema that resolved within hours, without evidence of barrier disruption. In contrast, the bMN site showed persistent erythema and punctate erosions at insertion points (Figure S24, Supporting Information). These results directly validate the chronic dermatological safety of the sMN in humans, extending the biocompatibility evidence beyond rodent models.

These findings demonstrate that the sMN achieves a key design goal in wearable bioelectronics: semi‐implantable device with minimal sensory burden, safe removal, and long‐term skin compatibility. This positions the sMN as a compelling alternative for applications where user comfort and cosmetic integrity are essential, such as in pediatric, geriatric, or consumer‐grade monitoring systems.

### 2.5 Histological Analysis of Inflammation and Tissue Integration

**Figure 5:**
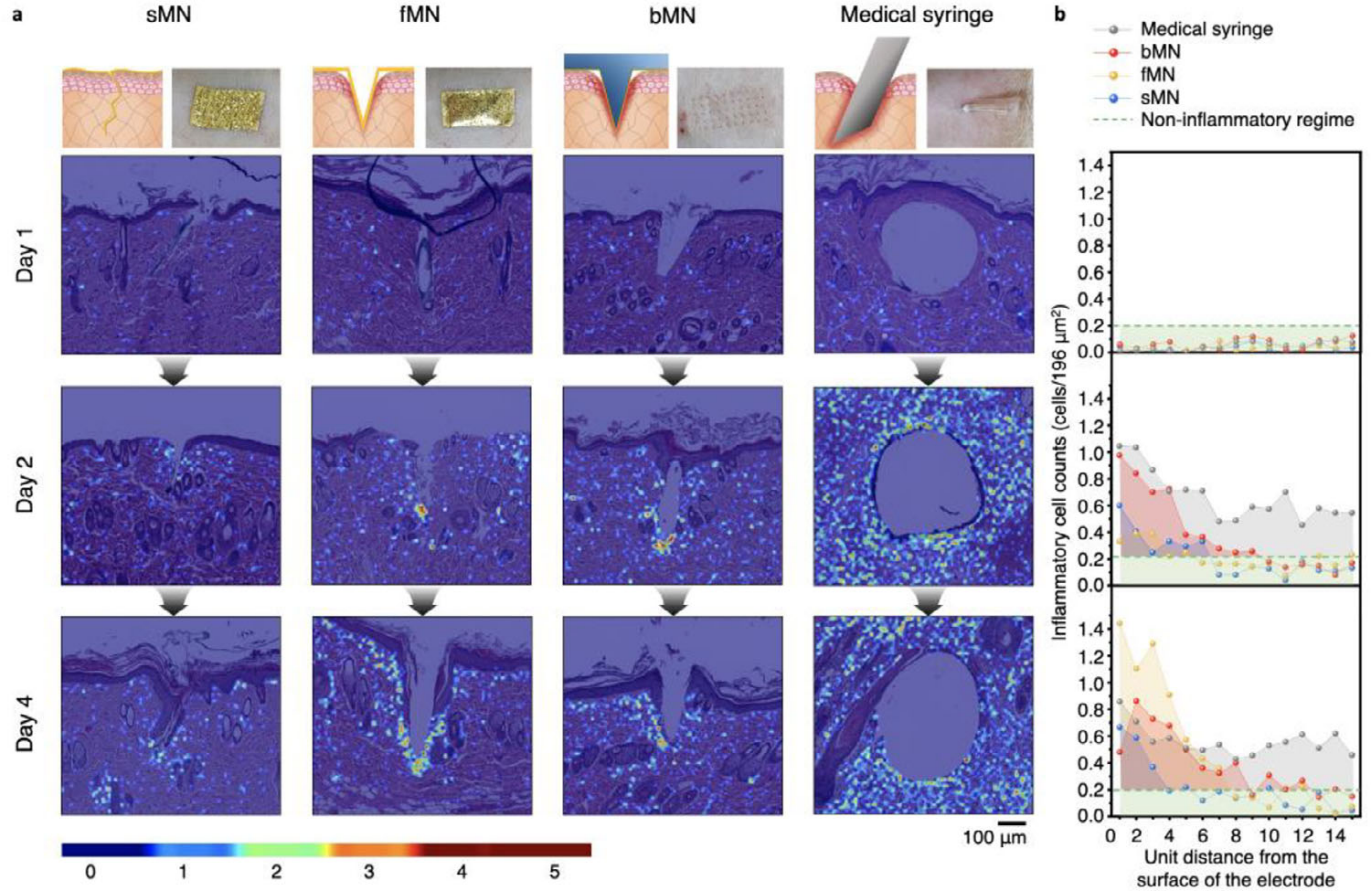
Histological mapping and quantitative analysis of inflammation induced by different percutaneous platforms. a), H&E‐stained tissue sections of SD rat skin implanted with sMN, fMN, bMN, or a 26G medical syringe, collected on days 1, 2, and 4 post‐insertion. Inflammation severity was visualized using 5‐level colormap overlays to highlight immune cell distribution around the insertion sites. b), Quantitative inflammatory cell counts plotted as a function of distance from the device–tissue interface. Dashed line indicates the threshold for “non‐inflammatory regime” (<0.2 cells per 196 µm^2^).

To evaluate tissue‐level responses and immune dynamics of the sMN electrode, we conducted histological analysis combined with immune cell‐type‐specific quantification (neutrophils, macrophages, lymphocytes) over 1–7 days post‐insertion in SD rat skin. Our primary analysis focused on comprehensive cell‐level quantification at Days 1, 2, and 4 for all device groups (sMN, fMN, bMN, syringe), supplemented by Day 7 H&E histology for sMN and bMN to assess long‐term immune resolution (**Figure** **5**a; Figure S25, Supporting Information).

The H&E images were further processed with 5‐level colormap overlays of immune cell density (Figure 5a; Figures S26 and S27, Supporting Information), enabling intuitive visualization of the spatial distribution and intensity of inflammatory responses.

Hematoxylin and eosin (H&E) staining at Days 1, 2, and 4 revealed distinct spatiotemporal inflammatory patterns for the four device types. The bMN and syringe groups induced strong, localized or diffuse inflammatory infiltrates, dominated by neutrophils and macrophages, whereas the fMN elicited moderate macrophage accumulation with limited neutrophil presence. The sMN consistently showed minimal immune activation with a balanced presence of macrophages and lymphocytes, suggesting a rapid transition toward resolution.

By Day 4, inflammatory areas in bMN and fMN expanded, reflecting ongoing mechanical stress from rigid microstructures. In contrast, sMN exhibited a reduced inflammatory footprint compared to Day 2, indicating effective suppression of immune propagation and minimal cellular stress. Quantitative cell‐type analysis (Figure S28, Supporting Information) confirmed that neutrophil presence was negligible in both sMN and fMN groups from Day 1 onward, whereas bMN retained neutrophils through Day 4. Syringe insertion caused a delayed but strong neutrophil surge at Day 2, while the sMN group showed an increasing macrophage‐to‐neutrophil ratio, indicative of early healing‐phase transition.

To characterize the spatial distribution of immune responses, we quantified immune cell density as a function of distance from the device–tissue interface (Figure 5b). Using a “non‐inflammatory regime” threshold of fewer than 0.2 immune cells per 196 µm^2^, we found that the inflammatory zone around sMN contracted from 7 unit distances (14 µm each) on Day 2 to 4 unit distances on Day 4, indicating rapid resolution. By contrast, fMN and bMN exhibited expanding inflammatory zones (6 to 8 and 10 to 13 units, respectively), while the syringe group showed persistent diffuse inflammation.

These findings collectively demonstrate that sMN effectively minimizes mechanical and cellular stress, guiding a faster transition from acute to chronic phases compared to other devices.

To further verify safe and residue‐free removal of sMNs, we performed histological analysis immediately after device extraction (Figure S26, Supporting Information). H&E staining confirmed the absence of residual fragments or acute tissue damage at the insertion site, consistent with our in situ Micro‐CT observations and finite element analysis (Figures S17 and S19, Supporting Information). The buckling and folding behavior observed in Figure 3 is a reversible structural adaptation, not material fracture, as validated by in situ retrieval imaging (Video S2, Supporting Information).

### 2.6 Long‐Term Chronic Immune Response

To examine later‐phase responses, we extended histological analysis to Day 7 for sMN and bMN, which represent the lowest and highest local inflammation profiles, respectively. Day 7 H&E results confirmed that sMN maintained negligible immune cell accumulation and intact tissue structure, validating that its minimal‐inflammatory profile is sustained over time. In contrast, bMN exhibited slightly intensified immune infiltration compared to Day 4, with ongoing neutrophil and macrophage activity, indicating incomplete resolution and persistent tissue stress (Figure S29, Supporting Information).

To further validate our histological quantification framework, we performed a TUNEL assay on bMN at Day 7 (Figure S30, Supporting Information), revealing pronounced apoptotic cell death in regions of persistent inflammation. This apoptosis profile corresponded closely with our temporal immune cell dynamics (neutrophils, macrophages, lymphocytes) measured across Days 1–4, providing independent confirmation that our cell‐level analysis accurately reflects tissue stress and recovery (Figure S31, Supporting Information).

Despite technical challenges (e.g., hair regrowth leading to patch detachment) that limited longer‐term evaluation beyond Day 7, the combined dataset (Days 1–4 cell profiling plus Day 7 validation) provides a coherent, biologically consistent view of acute‐to‐chronic immune transition, demonstrating early immune tolerance and resolution in soft MN designs (Figures S29 and S31, Supporting Information). These findings highlight that sMN's mechanical compliance and conformality prevent prolonged inflammation, enabling safe, long‐term bioelectronic integration.

### 2.7 Physical Decoupling of Biosignals From Environmental Noise Under Dynamic Skin Conditions

To validate the capability of the sMN bioelectrode to maintain signal fidelity under physiologically dynamic conditions, we assessed its performance in acquiring stable electrophysiological signals under varying environmental and temporal variations. A key limitation of conventional epidermal electrodes is their susceptibility to external noise arising from changes in skin hydration, such as sweating or dehydration. This results from an electrode positioned outside the SC, where the SC acts as a variable impedance barrier, modulating signal transmission based on local ionic mobility.

**Figure 6:**
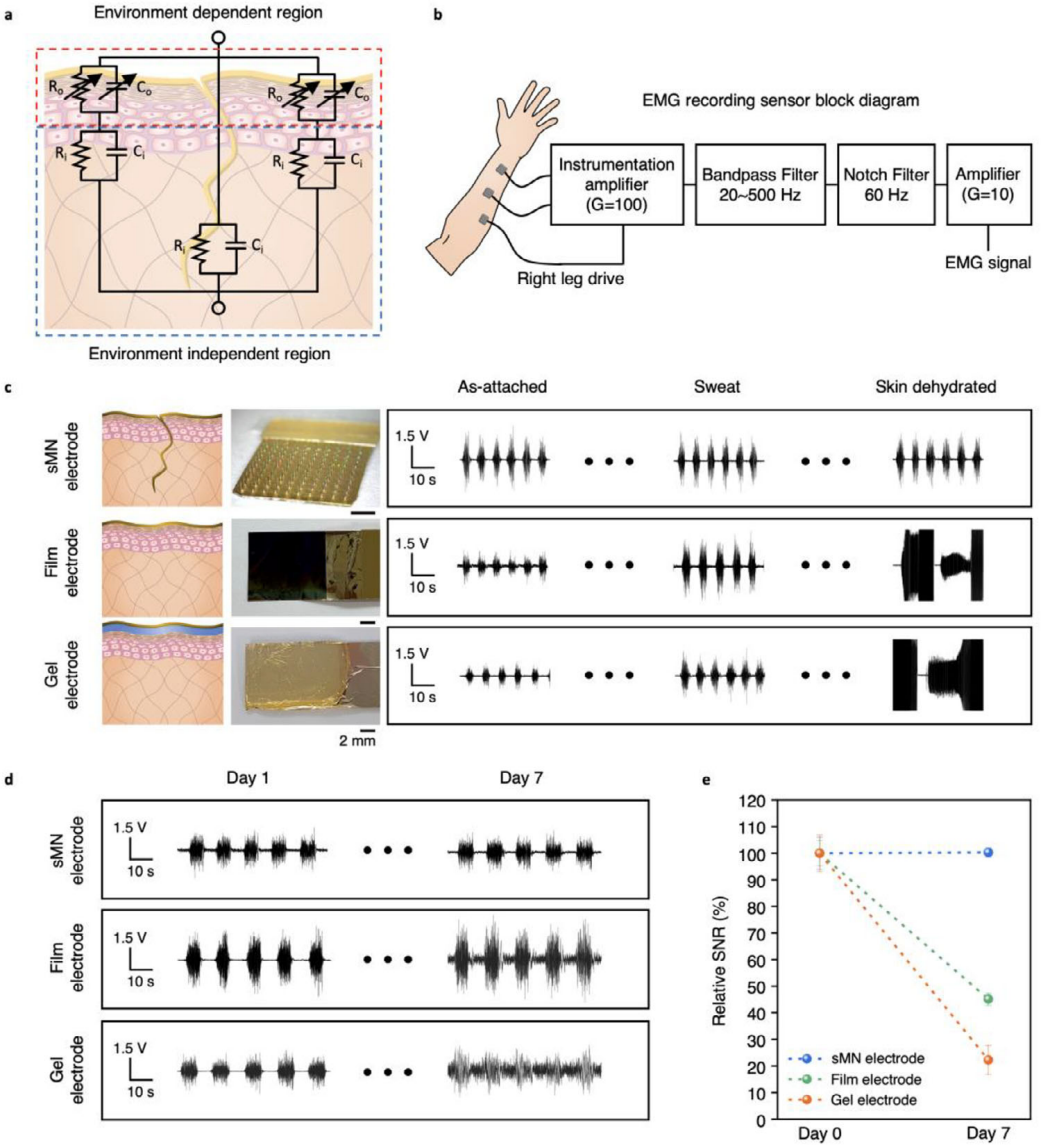
Long‐term and environment independent electrophysiological signal monitoring using sMN electrodes. a), Equivalent circuit model of the skin–electrode interface. The epidermis introduces variable impedance; the dermis maintains stable electrical properties. b), Block diagram of the EMG signal acquisition system. c), EMG waveforms recorded using sMN (top), film (middle), and gel (bottom) electrodes under wet and dry skin conditions. d), Longitudinal EMG recordings from each electrode type over 7 days. e), Relative SNR (%) of each electrode over time, rescaled to day 0 baseline.

We modeled this effect using an equivalent circuit diagram (**Figure** **6**a), where the epidermis is represented as a dynamic resistive‐capacitive (RC) interface and the dermis as a stable, low‐impedance layer. In conventional surface electrodes, fluctuations in hydration alter the RC properties of the SC, leading to inconsistent signal acquisition.^[^ ^41^, ^50^ ^]^ In contrast, the sMN bypasses the SC entirely, embedding its active sensing site into the dermis, thereby accessing an impedance‐invariant tissue environment. This design accesses a hydration‐invariant tissue environment with inherently lower impedance. Figure S32 (Supporting Information) describes the selective etching of the passivation layer and coating of conductive polymer on the MN tips.

For long‐term human EMG evaluations, we focused on film and gel electrodes as control groups, while bMN and fMN were excluded due to their insufficient skin compatibility during prolonged wear. Preliminary in vivo experiments confirmed that bMN and fMN cause localized microtrauma and inflammation after extended use, making them unsuitable for multi‐day continuous measurements. Short‐term testing indicated that bMN and fMN can provide comparable SNR to the sMN, but their long‐term instability and skin irritation risks preclude their use in human trials.

In addition to hydration‐dependent conditions, we investigated the impact of active skin deformation on EMG signal fidelity. Using both sMN and film electrodes, we performed EMG measurements while subjecting the skin to controlled stretching and compressive bending. The results revealed that EMG waveforms and amplitudes were identical to those recorded in undeformed states, confirming that electrode deformation did not compromise signal integrity. Furthermore, under voluntary movements, consistent EMG signals were recorded for both electrode types, and during involuntary or resting states, no spurious or noise signals were detected (Videos S5–S7, Supporting Information). These findings demonstrate that the conformal contact of both sMN and film electrodes effectively prevents motion‐induced artifacts, while the percutaneous interface of sMN provides additional stability over extended wear and environmental fluctuation.

Using a standard EMG acquisition system (Figure 6b; Figure S33, Supporting Information), we recorded muscle activity from the human forearm using sMN, film, and gel electrodes under both wet and dry conditions (Figure 6c). Only the sMN maintained a stable signal waveform and amplitude across all environmental states. In contrast, gel and film electrodes exhibited signal distortion and amplitude fluctuation under sweat and dehydration, highlighting their dependency on surface hydration for ionic conduction. Notably, the film and gel electrode occasionally failed to produce measurable EMG signals under dry conditions (relative humidity ≈20%) due to insufficient ionic conduction, occasionally behaving like an open‐circuit interface.

We next tested the long‐term signal stability of each electrode type by continuously monitoring EMG signals over 7 days (Figure 6d; Figures S34 and S35, Supporting Information). While the gel electrode dried out and the film electrode exhibited increasing impedance and signal degradation, the sMN preserved a consistent signal profile. This stability is attributed to its mechanically integrated percutaneous interface, which maintains electrical coupling even as the skin undergoes hydration cycles and minor movements.

To quantify this effect, we compared the signal‐to‐noise ratio (SNR) of each electrode across the monitoring period (Figure 6e). The sMN maintained over 100% of its initial SNR after 7 days, whereas the film and gel electrodes declined to 45% and 22%, respectively. The film electrode was fabricated using the same Parylene‐based dry interface as the sMN but lacks the percutaneous structure, while the gel electrode represents a conventional wet interface with hydrogel electrolyte, allowing a direct dry‐versus‐wet performance comparison. Consistent with these findings, we further extended the human evaluation window to 15 consecutive days (Figure S36, Supporting Information). Over this period, the sMN maintained remarkable reproducibility, with relative changes of only +0.33% in SNR, –5.46% in amplitude, and +2.17% in baseline noise compared to Day 1. Importantly, all values remained within the ±5–6% tolerance typically associated with biological variability in surface electrophysiology. These minimal drifts confirm that both signal quality and stability were preserved over two weeks of continuous use, providing direct evidence of the platform's robustness for chronic electrophysiological monitoring in practical human settings.

Finally, to demonstrate the practical utility of the newly developed cell‐stress‐free percutaneous bioelectrode, we validated its motion artifact‐independent performance through real‐time EMG recordings under both voluntary and involuntary muscle activity (Videos S5–S7, Supporting Information). Dynamic EMG recordings performed under controlled electrode deformation further confirmed that both sMN and film electrodes preserve signal quality during stretching or bending. Furthermore, dynamic EMG recordings during treadmill running further validated the stability of the sMN electrode, which maintained hydration‐insensitive signal quality compared to film and gel electrodes that exhibited sweat‐induced signal distortion (Figure S37 and Video S9, Supporting Information). Quantitative comparisons of SNR, baseline drift, and the magnitudes of both signal and noise during running highlight the sMN's practical robustness under motion and hydration. In addition, the bioelectrode was successfully integrated with commercially available real‐time biosignal acquisition platforms, confirming its compatibility and readiness for practical deployment (Video S8, Supporting Information). A summary evaluation table (Table S4, Supporting Information) highlights the relative advantages of the sMN platform over gel, film, and invasive electrodes, and clarifies the rationale for selecting film and gel electrodes as realistic long‐term benchmarks. This table outlines key performance metrics, including signal quality, biocompatibility, user comfort, conformality, long‐term usability, and fabrication complexity, and illustrates the unique balance achieved by the sMN electrode between minimally invasive integration and robust long‐term performance. While these results establish the superiority of sMN over the most relevant experimental controls (film and gel electrodes), a broader perspective requires benchmarking against the diverse range of wearable bioelectrodes reported previously. To this end, we extended our analysis from a qualitative summary to a quantitative, parameter‐based framework. Tables S5 and S6 (Supporting Information) define three orthogonal criteria—tissue compatibility, long‐term operation, and depth of signal origin—which were used to construct a 3D performance map (Figure S38, Supporting Information). This visualization situates each representative device within a standardized design space, clearly positioning the sMN at the high‐performance corner and underscoring its unique integration of mechanical compliance, stable operation, and selective percutaneous signal acquisition.

## 3 Conclusion

Our work presents a mechanically adaptive, percutaneous bioelectrode platform that resolves two major limitations of wearable biosensors: environmental signal degradation and chronic mechanical stress at the tissue interface. Through the use of an effervescent sacrificial scaffold and an ultrathin parylene‐C film, we developed a sMN that undergoes structural reconfiguration after insertion, transitioning from a stiff, penetration‐capable state to a soft, compliant one. This transformation enables elastic buckling rather than fracture under shear, a ≈99.7% mass reduction, and retention of electrical integrity even after 1000 deformation cycles. Volumetric compliance and omnidirectional displacement—up to 9 times greater than conventional MNs—allow the sMN to mechanically harmonize with dynamic skin movement, minimizing internal stress concentration. Combined with its percutaneous positioning in the dermis, the sMN offers stable electrical coupling across hydration states, as confirmed by environment‐independent characteristics. This feature collectively establishes the sMN as a continuous EMG acquisition for long‐term biosignal monitoring.

In addition to its mechanical advantages, the sMN exhibits exceptional biocompatibility and immunological tolerance. Human‐subject testing demonstrated minimal discomfort and no visible skin irritation after extended use, contrasting with the redness, pore size, and user‐reported pain caused by bulky and fMNs. Histological analysis in rat models revealed minimal tissue disruption and a substantially reduced inflammatory profile in the sMN group, even after four days of continuous implantation. The concept of a “non‐inflammatory regime”, defined by localized immune cell density, offers a quantifiable framework for assessing biocompatibility in MN interfaces. Notably, only the sMN decreased the distance required to reach the non‐inflammatory regime, but the fMN and bMN represented an opposite tendency, meanwhile, the syringe failed to approach the inflammatory threshold at any point. These results validate that cell‐level mechanical compliance translates directly into suppressed tissue‐level immune activation—an essential attribute for any bioelectronic device intended for chronic use.

While this study focused on electromyographic monitoring, the sMN's architectural and material design principles are broadly transferable to other bioelectronic modalities, including neural, cardiac, and metabolic sensing or transdermal drug delivery. The sMN's compatibility with commercial acquisition systems and wireless low‐power modules highlights its readiness for integration into practical wearable platforms. Moreover, its superior comfort, safety, and cosmetic invisibility make it suitable for vulnerable populations such as pediatric or geriatric patients, and for use in consumer‐grade health technologies. Just as the emergence of epidermal electronics a decade ago marked a transformative shift toward skin‐conformal devices, we propose that dermal electronics, as demonstrated here, represent the next paradigm. By relocating the interface from the unstable surface to the mechanically and electrically stable dermis, this work lays the foundation for a new generation of bioelectronic systems that combine long‐term signal fidelity, immune invisibility, and real‐world usability.

## 4 Experimental Section

### Materials and Reagents

All chemicals were used as received unless otherwise specified. Sodium chloride (NaCl), ethanol (99.9%), and polyvinyl pyrrolidone (PVP, Mw 360 and 55 kDa) were obtained from Sigma‐Aldrich. Citric acid (JUNSEI), sodium bicarbonate (NaHCO_3_), and phosphate‐buffered saline (PBS, 1X, pH 7.4; ThermoFisher Scientific) were used without further purification. Deionized water was prepared using a Milli‐Q system (Millipore). All reagents used in electrode fabrication were handled under ambient laboratory conditions unless noted otherwise.

### Ethical Approval

Human subject protocols were approved by the POSTECH Institutional Review Board (IRB No. PIRB‐2021_E064), with written informed consent obtained from all participants. Animal procedures were approved by the Institutional Animal Care and Use Committee (POSTECH IACUC No. 2023‐0129) and performed in accordance with institutional guidelines for the humane treatment of research animals.

### Fabrication of MN Patches

Master molds were fabricated by grayscale lithography using SU‐8 2150 (MicroChem Corp.) on 4‐inch glass wafers.^[^ ^30^, ^34^ ^]^ The resulting conical MN exhibited high aspect ratios with tip radii <42 µm, base diameters of 200 µm, heights of 774 µm, and 1 mm tip‐to‐tip spacing (Figure S1g, Supporting Information). Optical inspection and dimensional verification were performed using a Dino‐Lite Plus digital microscope. Inverse PDMS molds were cast from the master structures and cured at 60 °C overnight.

For the effervescent MN patches, a homogeneous solution was prepared containing 6.5% (w/v) PVP (360 kDa), 6.5% PVP (55 kDa), 4% citric acid, and 0.5% NaHCO_3_ in ethanol.^[^ ^42^ ^]^ ≈250 µL of the solution was poured onto the PDMS mold (molding area: 180 mm^2^) and placed under vacuum (−85 kPa) for 48 h at 22 °C and 30% relative humidity. A beaker with 50 mL of ethanol was co‐placed in the vacuum chamber to maintain vapor pressure and reduce air bubble entrapment during cavity filling. After solidification, rigidified patches were demolded and stored in vacuum desiccators until further use (Figure S1a–f, Supporting Information).

### Electrode Formation and Surface Modification

Effervescent MN patches were coated with 500 nm parylene‐C using a chemical vapor deposition system (PDS 2010, Specialty Coating Systems). To avoid backside coating, adhesive masking tape was applied before deposition. Gold electrodes (100 nm thick) were deposited on the parylene surface by thermal evaporation (TheONE SCIENCE) at 1 × 10^−^⁵ torr. A secondary 500 nm parylene‐C layer was deposited for electrical insulation, forming a sandwich structure (Figure S3a, Supporting Information).

To selectively expose the MN tips, reactive ion etching (RIE) with oxygen plasma was performed (Femto Science, CIONE; 200 W, 50 kHz, 100 sccm O_2_ flow, 7 × 10^−^^3^ torr, 7 min) using a parafilm shadow mask (100 µm circular openings) aligned to mid‐shaft. Exposed gold tips served as the electrode's active sites (Figure S32a–e, Supporting Information).

Conductive polymer deposition was performed to enhance interface conductivity. PEDOT:PSS was electrochemically deposited using a two‐electrode galvanostatic method, applying stepped currents of 0.2, 0.4, 0.6, and 0.8 µA for 150 s each, followed by 1 µA for 600 s. The MN electrode was used as the working electrode and a Pt mesh as the counter/reference electrode (Figure S32f,g, Supporting Information).

### Fabrication and Structure of bMN, sMN, and fMN

The microneedle (MN) structures were fabricated using a multilayer parylene deposition process combined with a sacrificial microneedle template.

The bMN consists of a sacrificial microneedle patch coated with a parylene–gold–parylene trilayer (500/100/500 nm). The sacrificial core, composed of polyvinylpyrrolidone (PVP) mixed with citric acid and sodium bicarbonate, provides the initial mechanical rigidity for skin insertion (Figure S39a, Supporting Information). The Au layer (100 nm) is selectively exposed at the tip to function as the electrode interface.

After insertion, a small amount of water is applied to the bMN, initiating an effervescent reaction of the sacrificial core (PVP–citric acid–sodium bicarbonate), which dissolves within ≈5 min and leaves only the ultrathin parylene–gold–parylene film (500/100/500 nm) as the final electrode structure (Figure S39b, Supporting Information). This thin‐film architecture exhibits high flexibility and conforms to skin curvature while maintaining electrical conductivity through the exposed Au interface.

The fMN is fabricated using a similar process but employs a 5 µm parylene base layer instead of a sacrificial core to create a semi‐rigid structure (Figure S39c, Supporting Information). The final configuration is a parylene–gold–parylene trilayer (5 µm/100 nm/5 µm), which is thicker and less flexible than the sMN but softer than the original bMN.

All parylene layers were deposited using chemical vapor deposition (CVD) with controlled thickness monitored by in situ quartz crystal microbalance. The Au layer was patterned by thermal or electron‐beam evaporation, followed by oxygen plasma etching to selectively expose the electrode tip.

### Mechanical and Electrical Characterization

To measure lateral compliance, single MNs from both sMN and bMN samples were subjected to shear force by displacing them laterally against a rigid surface while recording force‐displacement profiles (n = 5). All MNs were inspected under microscopy before and after deformation to assess plastic failure or cracking (Figure 2a, Figure 2c, middle, and Figures S6 and S7, Supporting Information).

To quantify insertion rigidity, individual sMNs were subjected to axial compression using a mechanical testing machine (Instron) equipped with a 10 kN load cell, with the force applied perpendicularly to the MN tip at a constant rate of 0.05 mm s^−1^. The fracture force was defined as the point of sudden force drop in the stress–strain curve. All tested sMNs exhibited fracture forces exceeding 0.05 N, the threshold commonly cited for reliable skin penetration (Figure S40, Supporting Information).

Insertion performance was further validated using optical coherence tomography (OCT). Arrays of 81 sMNs were applied to ex vivo porcine and SD rat skin using a thumb‐pressing method for 10 s. OCT cross‐sectional imaging confirmed complete and uniform insertion across the entire MN array, with clear signal voids penetrating through the stratum corneum into the upper dermis (Figure S41, Supporting Information).

Fatigue durability was evaluated by subjecting sMN electrodes to 180° bending cycles using a servo motor driven by an Arduino Leonardo R3. A copper wire was attached to the MN substrate to transmit the bending force. Bending occurred at 0.34 mm s^−1^, and impedance at 100 kHz was recorded using a digital multimeter after each cycle up to 1000 repetitions (Figure 2c, right, Figure S11, and Video S2, Supporting Information).

### Electrochemical Characterization (EIS and CV)

Electrochemical impedance spectroscopy (EIS) and cyclic voltammetry (CV) measurements were performed using an Electrochemical analyzer (Model 2101A, Solatron Analytical Inc.). A three‐electrode configuration was employed, with the sMN array as the working electrode, a platinum wire as the counter electrode, and an Ag/AgCl (3 M KCl) electrode as the reference. All measurements were conducted in 1× phosphate‐buffered saline (PBS 1x, pH 7.4) at room temperature (∼25 °C).

For EIS, the frequency was swept from 100 Hz to 100 kHz with an AC perturbation amplitude of 10 mV (rms) at open‐circuit potential. Impedance values at 100 Hz, 1, 10, and 100 kHz were extracted for comparison.

For CV, scans were conducted over a potential window of 0 V to +1.2 V versus Ag/AgCl at a scan rate of 50 mV s^−1^, with at least three consecutive cycles recorded to confirm stability.

The effective electrode area was standardized to 1 cm × 1 cm for all samples. All electrodes were rinsed with deionized water and dried with nitrogen before measurements to ensure reproducibility.

### Imaging and Visualization

X‐ray imaging of MNs inserted into *ex vivo* SD rat skin was conducted at the Pohang Light Source‐II (PLS‐II) synchrotron facility. 2D and 3D imaging was performed using a white‐beam X‐ray generated by a bending magnet‐based beamline. Samples were placed on both fixed and rotating stages to capture planar and tomographic datasets, respectively (Figure 3a,b; Figures S12 and S13, Supporting Information).

Projection data were collected using a PCO Edge 5.5 camera (pixel size: 6.5 µm; effective resolution: 0.65 µm with 10× lens). A dataset of 2560 × 2560 × 700 voxels was reconstructed using a custom MATLAB software (PHOVIS), employing CUDA‐accelerated volume rendering. All images were downsampled 1:2 for storage and real‐time rendering (Figure 2b; Figure S14, and Video S3, Supporting Information).

SEM imaging was performed after Au sputtering using a magnetron coating unit (MCM‐100, SEC Inc.). Samples were imaged on an SNE‐4000 m SEM system (SEC Inc.) under 10–15 kV acceleration voltage (Figure S2, Supporting Information).

### Human‐Subject Skin Irritation and Discomfort Testing

16 healthy adults (25–32 years) participated in a blind discomfort evaluation comparing sMN, fMN, bMN, and 1 µm‐thick thin‐film patches. Each 5 × 5 mm^2^ device was randomly applied to the volar forearm. After 1 h, participants rated discomfort, including itching, tightness, or pain on a 0–10 visual analogue scale (VAS) (Figure 4a; Figure S20, Supporting Information).

For dermatological compatibility, a 31‐year‐old male subject wore 10 × 5 mm^2^ devices on the forearm for 7 days. A polyester cleanroom wiper (WW‐2209, KM Corporation) and Tegaderm (3 m) film provided water resistance and adhesive coverage. Skin redness and micropore visibility were imaged at baseline, immediately post‐removal, and 1 and 7 days post‐removal using a Dino‐Lite Plus microscope under constant lighting. Red pixel area and micropore diameter were analyzed with ImageJ. Evidence of dermatitis or scarring was also recorded (Figure 4b–d).

### In vivo Histology and Inflammation Quantification

Seven‐week‐old male SD rats were used for histological analysis. Devices (sMN, fMN, bMN, and 21G syringe) were inserted into shaved dorsal skin using a spring‐loaded applicator. Skin flaps were used to prevent patch displacement during movement (Figure S25, Supporting Information).

At 0, 1, and 3 days post‐insertion, skin samples were excised, fixed in 10% buffered formalin, paraffin‐embedded, sectioned at 4 µm thickness, and stained with hematoxylin and eosin (H&E). A certified pathologist annotated and labeled immune cells by type: macrophages (green), lymphocytes (blue), and neutrophils (red) (Figure S26, Supporting Information).

The region of interest (ROI) was centered on the MN insertion axis, and adjacent tissue was segmented into 14 × 14 µm^2^ grids, extending 15 units from the electrode‐tissue interface (Figure S27, Supporting Information). Inflammatory cell density was mapped in MATLAB using a 4‐color jet colormap ranging from dark blue (0 cells) to dark red (>4 cells) (Figure 5a).

Colormap code used for visualization of inflammatory cell density: resize_num = 100; zMin = 0; zMax = 4; data = load(“data_name.txt”); data = imresize(data, resize_num); Figure 1; imshow(data,[]);colormap jet; colorbar; clim([zMin, zMax]);

### Cytotoxicity Test In Vitro

To evaluate the cytotoxicity of the effervescent sacrificial materials, eluates were prepared by dissolving the dried citric acid and sodium bicarbonate mixture in deionized water, followed by the addition of polyvinylpyrrolidone (PVP) to mimic the formulation used in the device. The solution was filtered and diluted in cell culture medium to a final concentration of 5% (v/v). L929 fibroblast cells were seeded in 96‐well plates at a density of 1.5 × 10⁴ cells per well and incubated overnight. The culture medium was then replaced with the eluate‐containing medium, and cells were further incubated for 24 and 48 h. Cell viability was assessed using the Cell Counting Kit‐8 (CCK‐8, Dojindo), with absorbance measured at 450 nm after 1 h of incubation. Eluate‐treated cells exhibited viability comparable to the negative control (untreated wells), indicating no measurable cytotoxicity under the tested conditions (Figure S5, Supporting Information).

### TUNEL Assay for Apoptotic Cell Detection

To assess device‐induced apoptotic cell death in the tissue surrounding the insertion sites, we performed TUNEL (Terminal deoxynucleotidyl transferase dUTP Nick‐End Labeling) staining using the In Situ Cell Death Detection Kit, Fluorescein (Roche, Cat. No. 11 684 795 910), following the manufacturer's protocol.

Paraffin‐embedded skin tissue sections harvested at Day 7 post‐insertion from the bMN group were deparaffinized, rehydrated, and subjected to proteinase K treatment (20 µg mL^−1^ for 15 min at room temperature) to facilitate probe penetration. Sections were then incubated with the TUNEL reaction mixture for 60 min at 37 °C in a humidified chamber, followed by DAPI counterstaining to visualize cell nuclei.

Fluorescence images were captured using a confocal microscope (Zeiss LSM 800), and TUNEL‐positive apoptotic cells were quantified in the tissue regions adjacent to the insertion tract. Negative controls were processed without the terminal transferase enzyme to confirm signal specificity (Figure S30, Supporting Information).

### EMG Signal Acquisition and Environmental Testing

Electrophysiological recordings were conducted using a custom circuit containing an INA333 instrumentation amplifier and TLV9062 op‐amp (Texas Instruments). The system applied bandpass filtering (20–500 Hz), 60 Hz notch filtering, and total gain of ≈1000 times. A three‐electrode configuration (working, counter, reference) was used, with sMN, film, or gel electrodes applied to the forearm (Figure 6b; Figure S33, Supporting Information).

To test environmental interference, skin was subjected to dry air or PBS application to simulate dehydration and perspiration, respectively. Electrodes were placed on the same arm under randomized locations, and EMG signals were recorded during alternating fist contractions (Figure 6c).

For long‐term testing, identical electrodes were worn for 7 days under Tegaderm protection, followed by repeated EMG measurement. Signal stability was quantified by comparing SNR values at day 0 and day 7 (Figure 6d,e; Figures S34 and S35).

Motion artifact testing involved involuntarily moving the subject's forearm and wrist during EMG acquisition, with signals compared to those from active voluntary contractions. Commercial gel electrodes (3 m) served as reference throughout all trials (Videos S5–S7, Supporting Information).

### Fabrication of Film and Gel Electrode

Film electrodes were fabricated using a multilayer parylene–gold–parylene structure (500 nm/100 nm/500 nm).

1. A 500 nm parylene layer was first deposited onto a glass slide, followed by the deposition of a 100 nm gold layer.
2. The parylene/gold/parylene film was peeled off from the glass substrate and cut into strips (1 cm × 3 cm).
3. Electrical wiring was attached, and the entire sample was coated with an additional 500 nm parylene layer.
4. To expose the active electrode area (1 cm × 1 cm), a shadow mask was applied, and the film was etched.
5. The exposed gold surface was coated with PEDOT:PSS using a stepwise electrochemical deposition (20, 40, 60, and 80 µA for 150 s each, followed by 100 µA for 600 s) to achieve a low‐impedance interface.

Gel electrodes shared the same parylene–gold–parylene base structure (500/100/500 nm) as the film electrodes up to the wiring and parylene overcoating step.

1. The active electrode area (1 cm × 1 cm) was exposed via shadow mask and etching.
2. A 2 mm thick agar gel layer (2.65 wt.% in PBS 1x) was cast on the exposed gold surface and allowed to solidify in a refrigerator.

The agar gel was prepared by dissolving agarose powder in PBS (0.0265 g mL^−1^) using repeated heating cycles (microwave for 30 s, intermittent mixing, and cooling) until fully melted and homogeneous.

## Conflict of Interest

The authors declare no conflict of interest.

## Author Contributions

J.L. and G.Y. contributed equally to this work. The conceptualization of the study was led by J.L. The methodology was developed by J.L. and G.Y., with experiments designed by both and performed primarily by G.Y. The wireless data transfer experiment was conducted by J.‐H.B. Bioactive material synthesis was carried out by J.L. and G.Y., who also fabricated the microneedle master mould. The investigation involved contributions from J.L., G.Y., J.J., T.S.H., S.W.K., H.W.L., P.T.L., H.L., S.‐M.P., and G.L. Visualization was performed by J.L., G.Y., J.J., J.P., and C.K. Funding acquisition was managed by G.L., while project administration was handled by J.L. The study was supervised by J.L. and G.L. The original draft of the manuscript was written by J.L., G.Y., J.‐H.B., J.P., S.‐M.P., and G.L., with review and editing conducted by J.L. and G.Y.

## Supporting information



Supporting Information

Supplemental Video

## Data Availability

The data that support the findings of this study are available in the supplementary material of this article.
