# Supplementary material for: Cell‐Stress‐Free Percutaneous Bioelectrodes
Source: Adv Mater. 2025 Sep 9;38(5):e09719. doi: 10.1002/adma.202509719 (PMC12822530; doi:10.1002/adma.202509719)
Supplement: Supplementary file 1 — Supporting Information [file ADMA-38-e09719-s002.pdf]

ADVANCED MATERIALS

Supporting Information

for *Adv. Mater.*, DOI 10.1002/adma.202509719

Cell-Stress-Free Percutaneous Bioelectrodes

*Jungho Lee, Gaeun Yun, Juhyeong Jeon, Phuong Thao Le, Tae Sik Hwang, Jeongwoo Park, Jin-Hyeok Baek, Seung Whan Kim, Hyoun Wook Lee, Kisang Kwon, Jihee Kim, Hoon Lim, Chulhong Kim, Sung-Min Park and Geunbae Lim**

Supplementary Information

Cell-stress-free percutaneous bioelectrodes

Jungho Lee[1,2]†, Gaeun Yun[1]†, Juhyeong Jeon[1], Phuong Thao Le[3], Tae Sik Hwang[4], Jeongwoo Park[5,6], Jin-Hyeok Baek[3], Seung Whan Kim[2], Hyoun Wook Lee[7], Kisang Kwon[8], Jihee Kim[9,10], Hoon Lim[11], Chulhong Kim[1,5,3,12], Sung-Min Park[1,5,3,12], Geunbae Lim[1,3]*

[1]Department of Mechanical Engineering, Pohang University of Science and Technology, Pohang, 37673, South Korea

[2]Department of Emergency Medicine, College of Medicine, Chungnam National University, Daejeon, 34134, South Korea

[3]Division of Interdisciplinary Bioscience and Bioengineering, Pohang University of Science and Technology, Pohang, 37673, South Korea

[4]Department of Emergency Medicine, Yongin Severance Hospital; Yongin, 16995, South Korea

[5]Department of Electrical Engineering, Pohang University of Science and Technology, Pohang, 37673, South Korea

[6]Department of Biomedical Convergence Science and Technology, Kyungpook National University, Daegu 41566, Republic of Korea

[7]Department of Pathology, Samsung Changwon Hospital, Sungkyunkwan University School of Medicine, Changwon, 51353, South Korea

[8]Department of Clinical Laboratory Science, Wonkwang Health Science University, Iksan, 54538, South Korea

[9]Department of Dermatology, Yongin Severance Hospital; Yongin, 16995, South Korea

[10]Cutaneous Biology Research Institute, Yonsei University College of Medicine; Seoul, 03722, South Korea

[11]Department of Emergency Medicine, Soonchunhyang University Bucheon Hospital, Bucheon, 14584, South Korea

[12]Department of Convergence IT Engineering, Pohang University of Science and Technology, Pohang, 37673, South Korea

†These authors contributed equally to this work

*Corresponding authors: limmems@postech.ac.kr

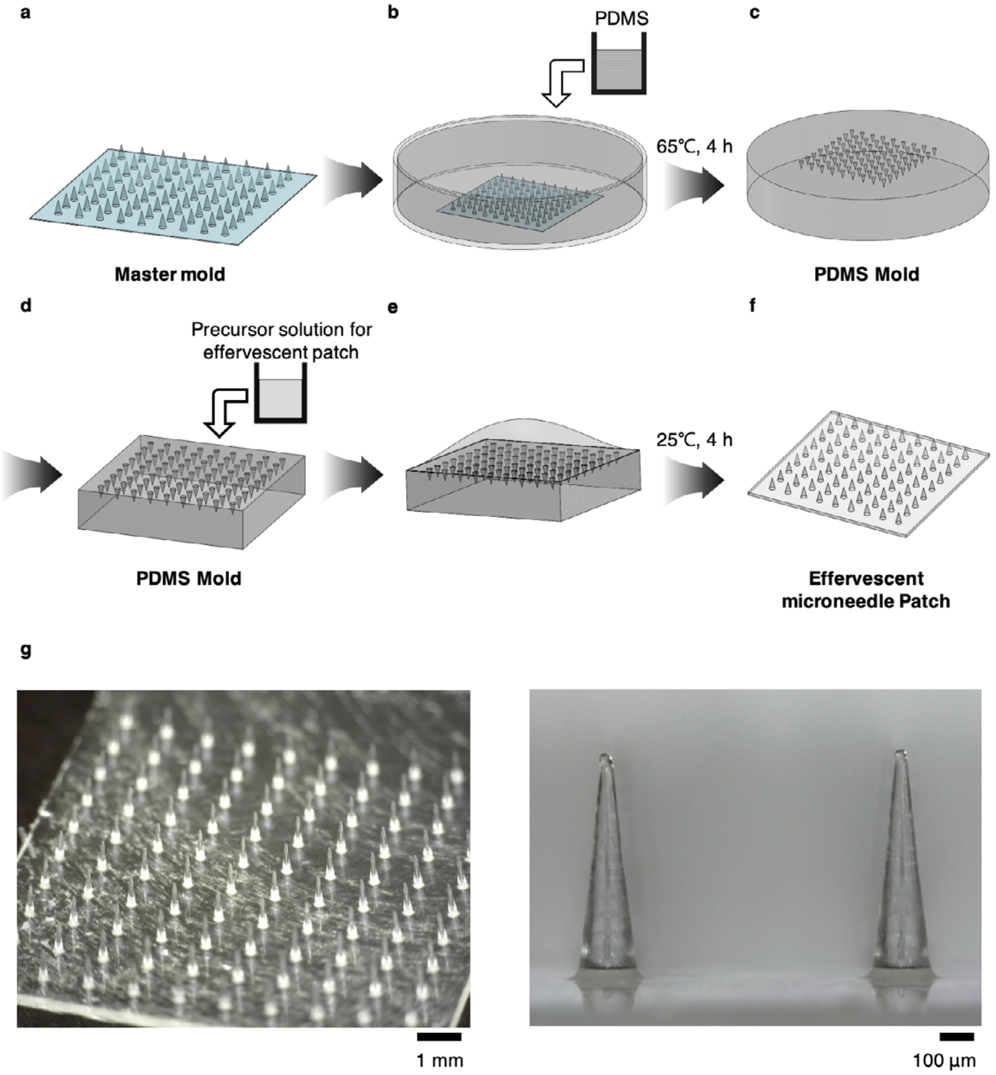

Supplementary Fig. 1 | Fabrication procedure of effervescent sacrificial MN patch. An MN array master mold was replicated via soft lithography using an effervescent sacrificial matrix containing polyvinylpyrrolidone (PVP), sodium bicarbonate ($NaHCO_3$), and citric acid. **a**, The MN master mold was prepared using grayscale lithography; detailed structure and fabrication procedures are described in the Methods section. **b**, The MN master mold was used for mold casting with polydimethylsiloxane (PDMS) to obtain a reversed mold. **c**, The PDMS mold was cured under 65℃ for 4 h. **d**, The precursor solution for the effervescent MN patch was poured onto the PDMS mold. **e**, The solution was vacuumed under 25℃ for 4 h to completely fill the MN cavities and dry the solution. **f**, The resultant effervescent MN patch was detached from the mold. **g**, Optical microscopy image of an effervescent MN patch; magnified MNs (left) and a whole MN patch (right). The MN has a height of 774 μm, a base diameter of 200 μm, a tip angle of 17° (<20°), a tip radius of 42 μm (<50 μm), and the pitch between MNs = 1 mm.

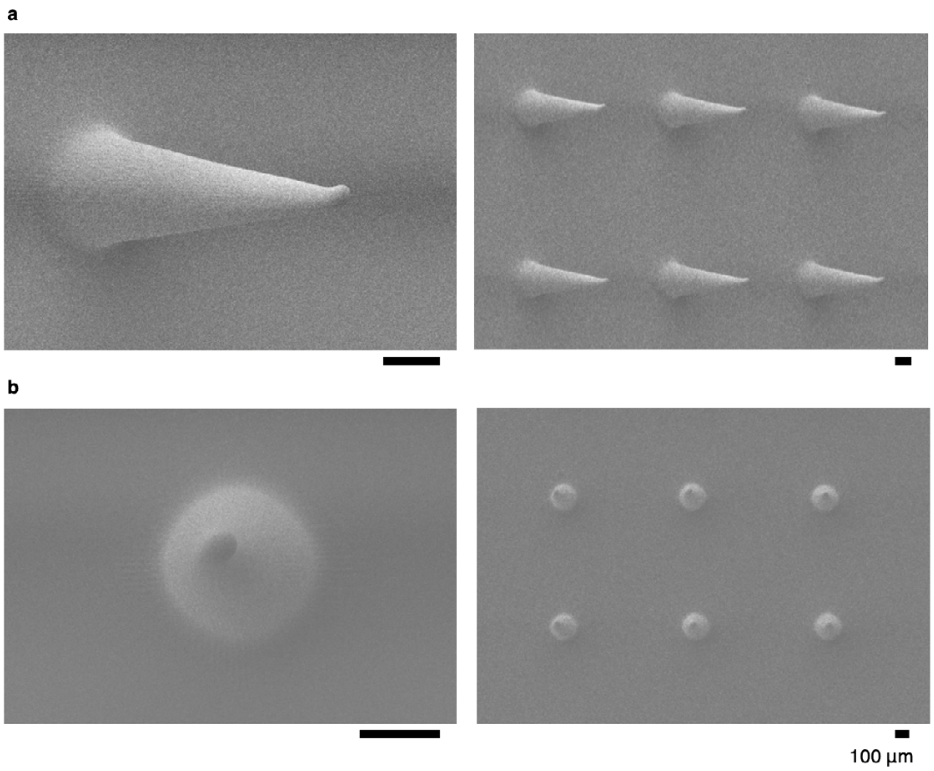

Supplementary Fig. 2 | Scanning electron microscopy (SEM) image of an effervescent sacrificial MN patch. a, Tilted view of a single MN (left) and a MN array (right). **b**, Top view of a single MN (left) and a MN array (right).

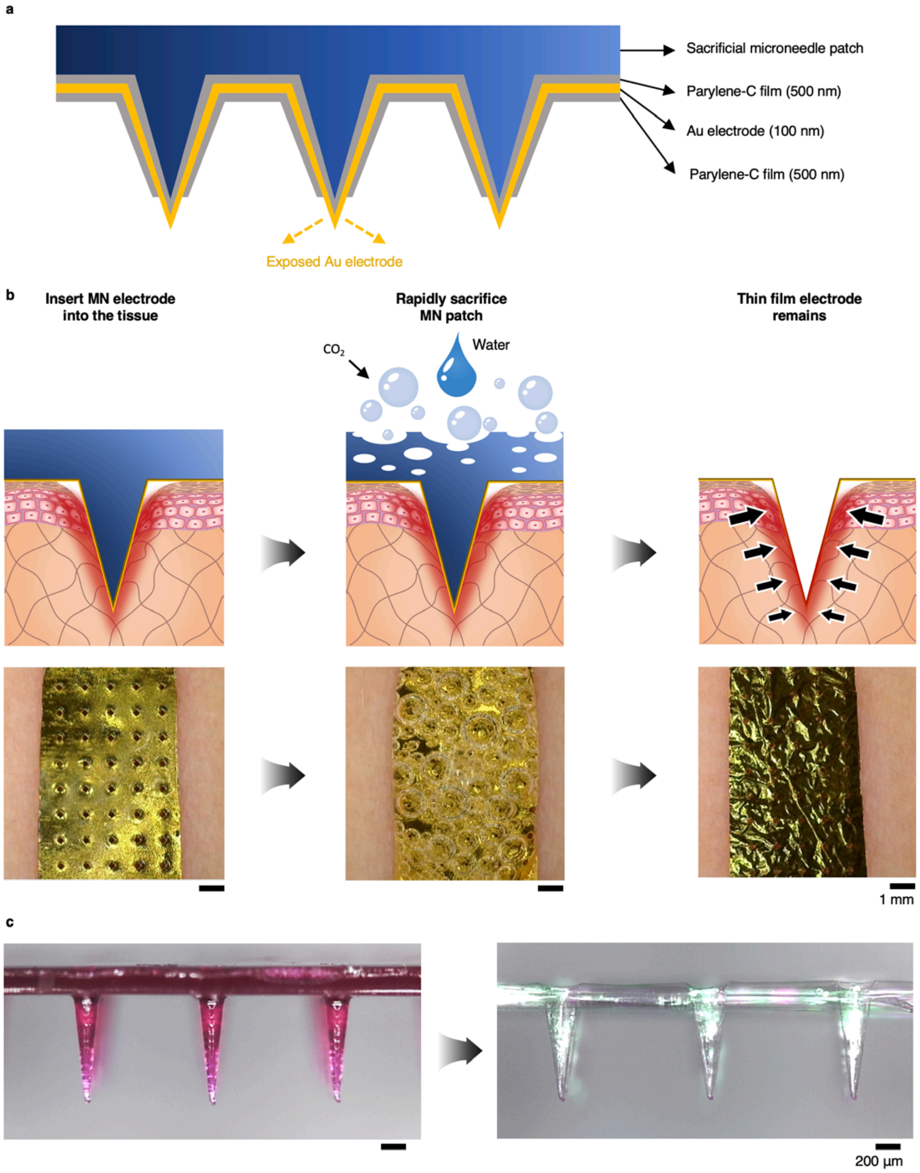

Supplementary Fig. 3 | Rapid dissolution of the effervescent microneedle matrix. a, Thin-film electrode has a layered structure: a 100 nm-thick thin-film Au electrode is sandwiched between 500 nm-thick thin-films of parylene-C for electric and ionic insulation, while only the tip of microneedles (MNs) are exposed to the Au electrode. The layered thin-film electrode is laminated on the surface of an effervescent MN patch. **b**, Time-lapse photographic sequence showing gas generation and sacrificial matrix disruption. **c**, Visualization of effervescent materials using a fluorescent dye. Fluorescent dye-containing the effervescent MN patch coated with the parylene (500 nm-thick) thin film (left) and a free-standing MN patch composed of a thin parylene film showing no residue of the fluorescent dye after removing the effervescent sacrificial MN patch (right).

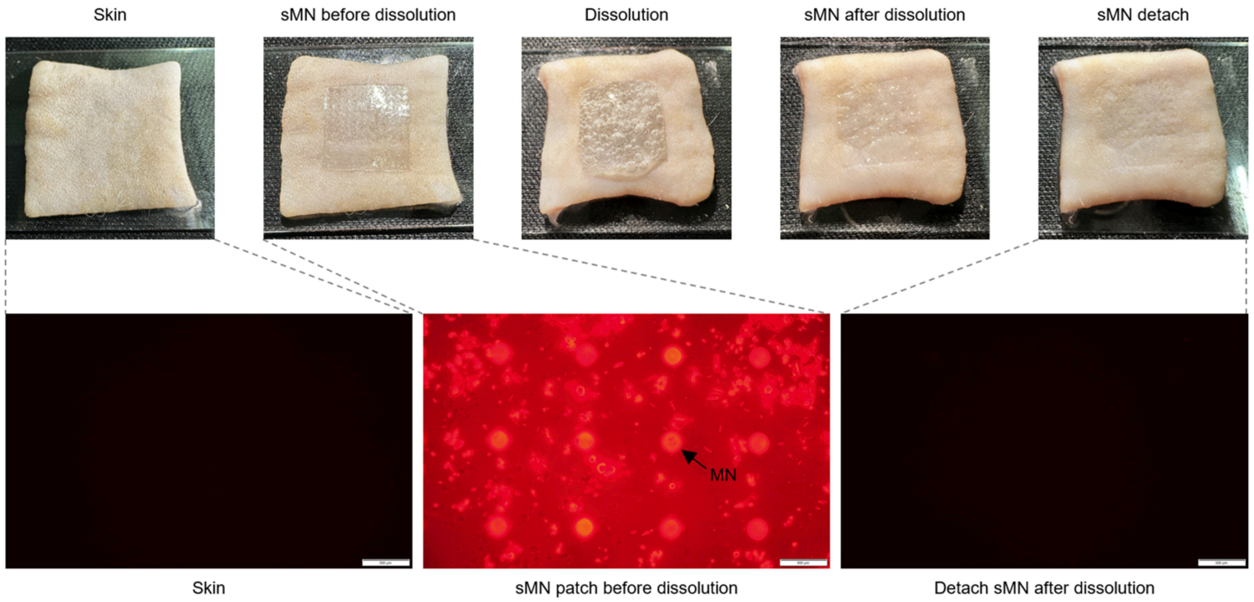

Supplementary Fig. 4 | Verification of residue-free dissolution of the sacrificial microneedle core. Fluorescently labeled effervescent MN patches coated with parylene thin layer were inserted into SD rat skin and monitored before and after the effervescent reaction. Fluorescence imaging confirmed that, after core dissolution, no residual fluorescent material was detected on or within the skin, demonstrating the residue-free and biocompatible nature of the effervescent removal process.

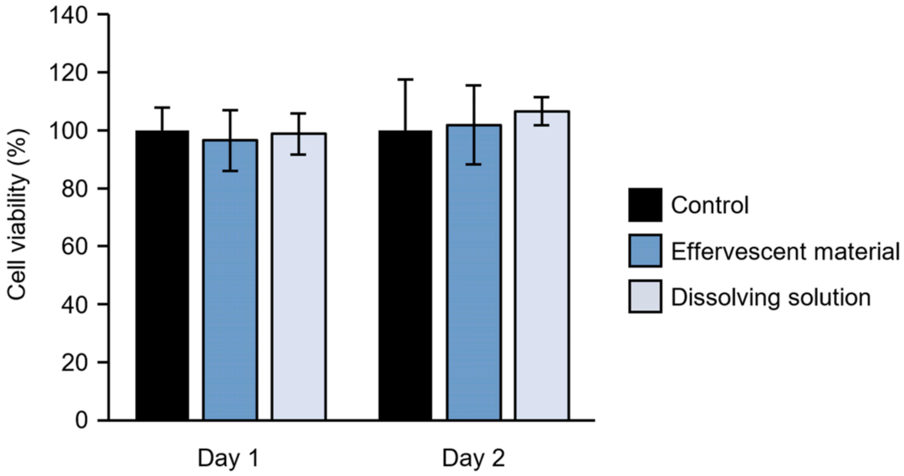

Supplementary Fig. 5 | Cytotoxicity assessment of effervescent materials and dissolving solution. Cell viability of L929 fibroblasts after 24 h and 48 h incubation with eluates from the effervescent material and dissolving solution compared to the control group. No significant reduction in viability was observed ($p > 0.05$), confirming that neither the effervescent components nor the dissolution process induces cytotoxic effects. ($n = 8$).

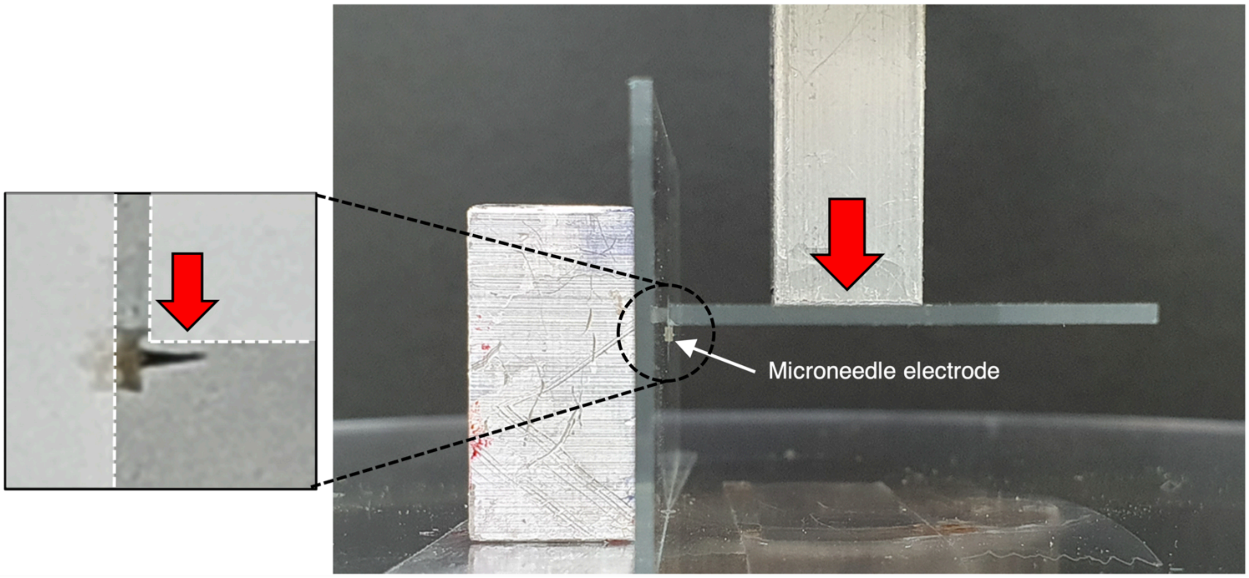

Supplementary Fig. 6 | Experimental setup for the measurement of mechanical compliance of a single MN electrode. A single MN is attached to the side wall. The load cell of the universal testing machine applies a force along the shear direction of the MN patch.

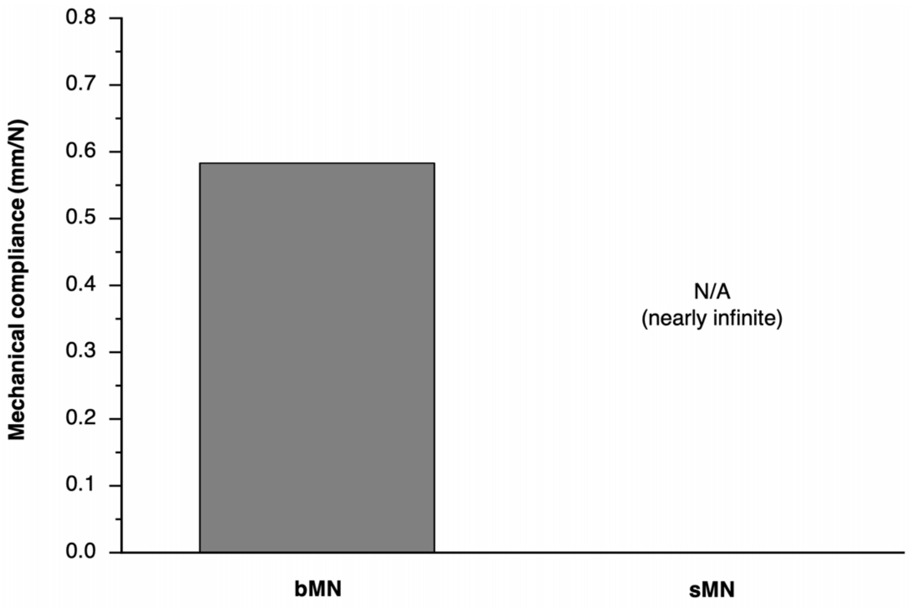

Supplementary Fig. 7 | Mechanical compliance of bMN and sMN. Mechanical compliance was calculated based on the load–displacement curve. While the bMN exhibited a compliance of 0.583 mm/N, the sMN showed nearly infinite compliance because the force required for measurement was below the sensing resolution of the universal testing machine.

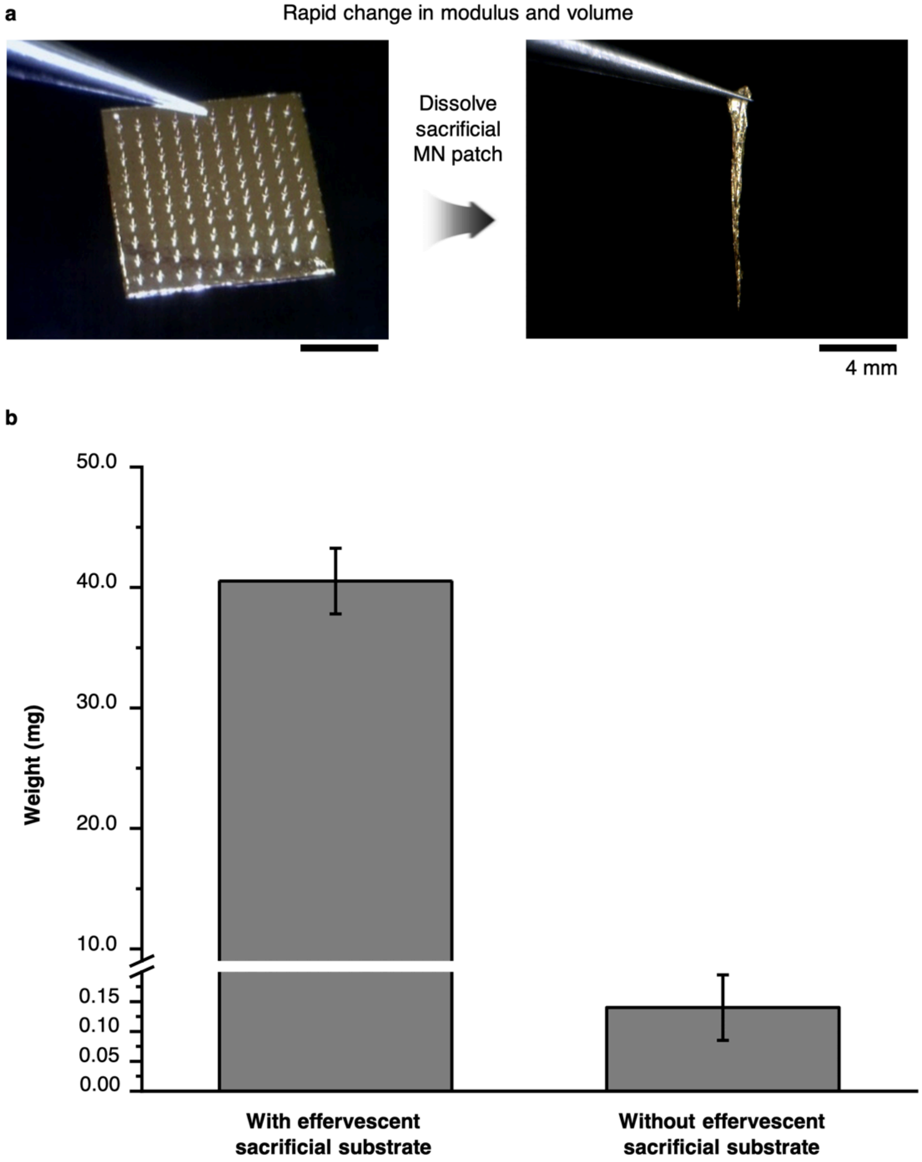

Supplementary Fig. 8 | Mass reduction after removing effervescent sacrificial substrate. a, Modulus and volume change of the MN patch. **b**, The removal of the sacrificial substrate decreased the weight of a bulky MN patch (size of the patch = 10 mm × 10 mm × 0.27 mm, number of MNs = 100) containing an effervescent sacrificial substrate by ~99.7% (from 40.54 mg to 0.14 mg).

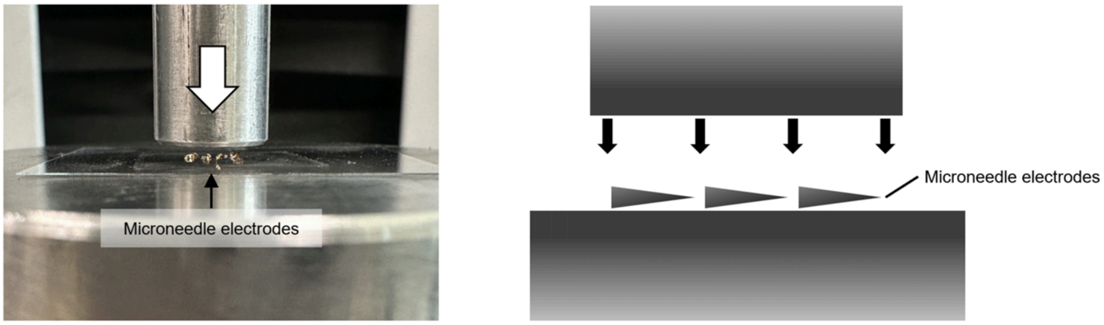

Supplementary Fig. 9 | Measurement setup for determining the modulus of the sMN electrodes. To evaluate the effective modulus of the sMN, 10 individual microneedles were horizontally arranged and compressed using a mechanical testing machine at a displacement rate of 0.04 mm/min. (Left) Photograph of the compression test setup. (Right) Schematic illustration of the uniaxial compression applied to the microneedle electrodes.

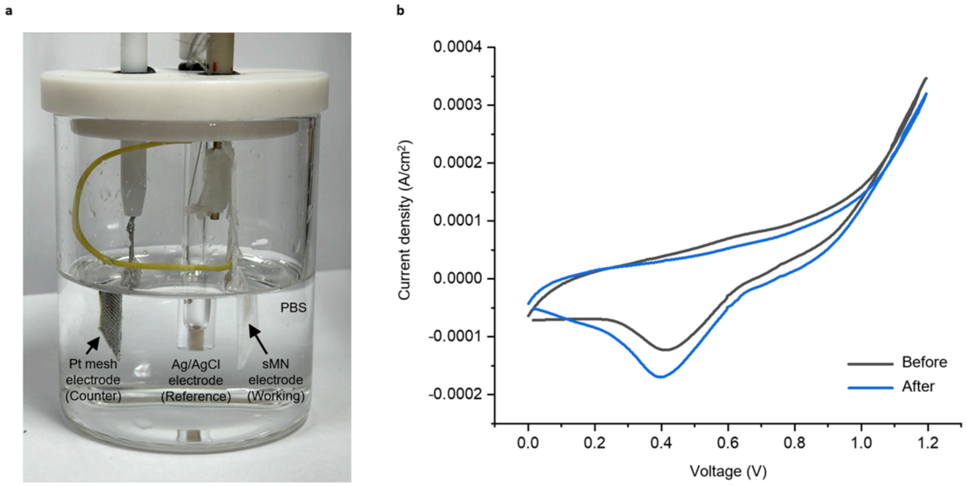

Supplementary Fig. 10 | Electrochemical characterization of the sMN electrodes under mechanical deformation. a, Three-electrode system used for electrochemical testing, consisting of the sMN electrode (working), Ag/AgCl (reference), and a Pt mesh electrode (counter) in PBS 1x solution. **b**, Cyclic voltammetry (CV) curves of the sMN electrode before and after 100 cycles of mechanical deformation (n = 7 for each condition). The CV profiles remain largely stable, confirming the robustness of the electrode interface. Minor variations arise from slight structural changes of the highly flexible sMN during repeated bending, which naturally affect the measured current density.

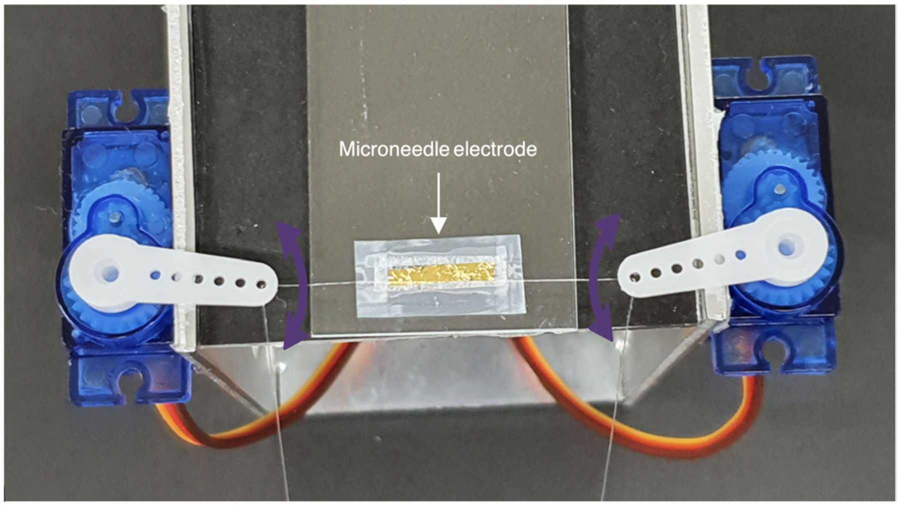

Supplementary Fig. 11 | Experimental setup for mechanical durability test. A row of MN electrodes is repetitively bent against the surface back and forth by 180°. A tensioned wire sweeps across the lower part of the MN electrodes to verify structural integrity.

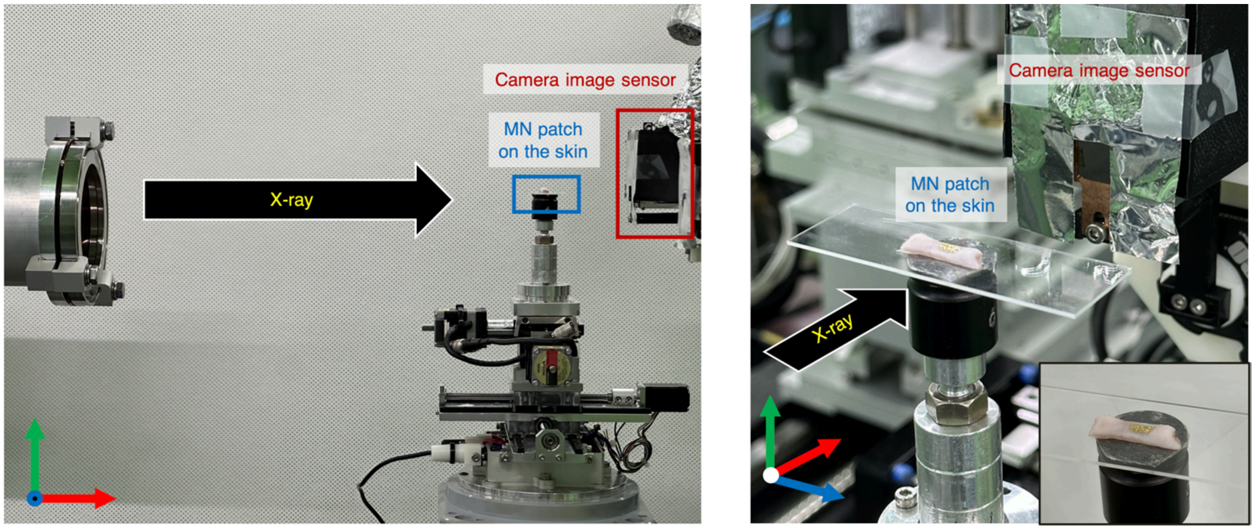

Supplementary Fig. 12 | Experimental setup for X-ray imaging. An MN patch applied onto the *ex vivo* SD rat skin (size: 5 mm × 15 mm × 3 mm) was placed on a stage, with X-rays projected parallel to the skin.

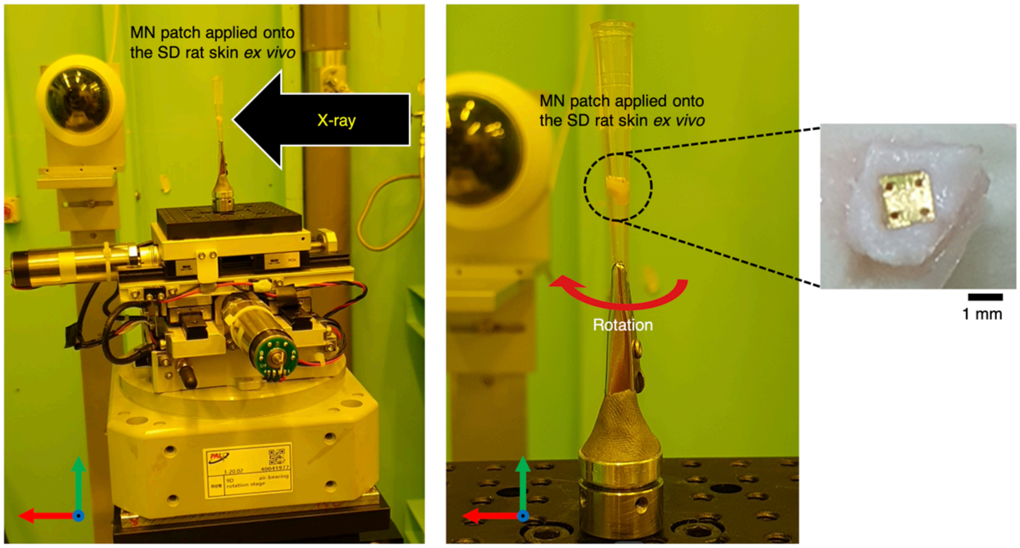

Supplementary Fig. 13 | Experimental setup for micro-CT 3D imaging. An MN patch applied onto the *ex vivo* SD rat skin (size: 3 mm × 3 mm × 3 mm) was placed on a rotating stage, with X-rays projected parallel to the skin.

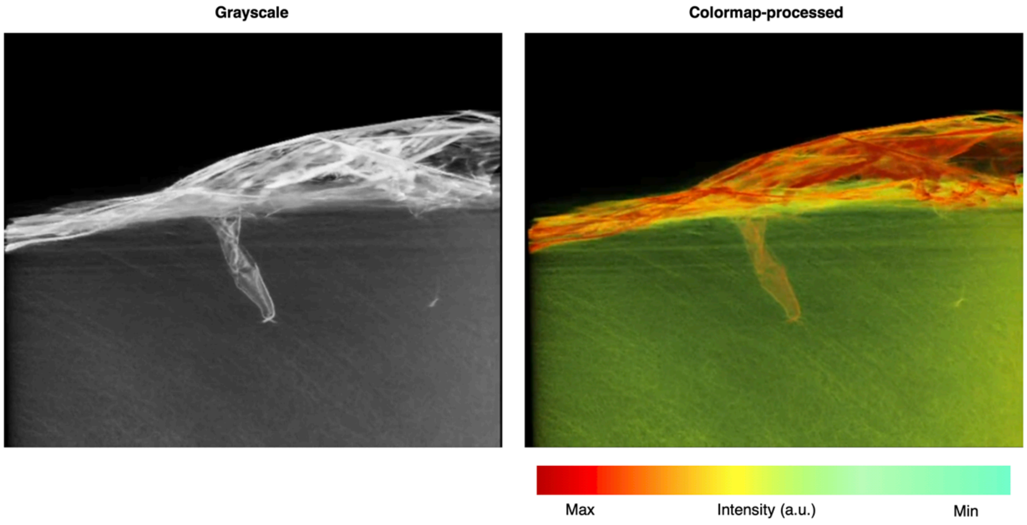

Supplementary Fig. 14 | Contrast-based colormap processing of an X-ray image of the sMN. Based on the contrast difference between the sMN and the skin, the original grayscale image (left) was converted into a colormap image (right), in which the sMN and skin are distinguished by color (red for sMN, green for skin). The color bar indicates the transition from green to red according to intensity.

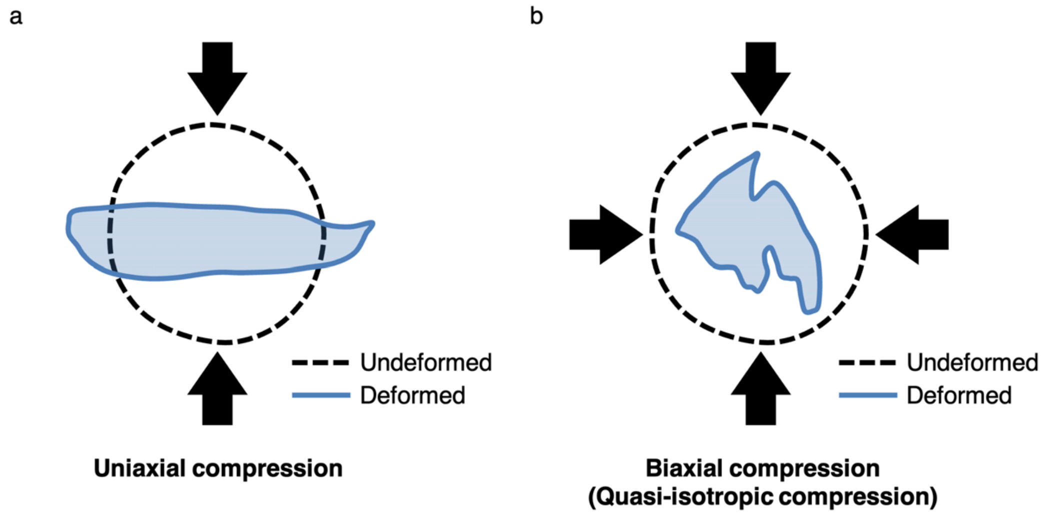

Supplementary Fig. 15 | Micro-CT-based visualization of sMN deformation modes under compression. a, Cross-sectional image of sMN under uniaxial compression, reconstructed from micro-CT data, showing lateral bulging (blue solid line) compared to the undeformed state (black dashed line), consistent with positive Poisson's ratio behavior. **b**, Cross-sectional image of sMN under biaxial compression, also derived from micro-CT, highlighting internal collapse with minimal lateral expansion, effectively mimicking a near-zero Poisson's response by redistributing volumetric strain.

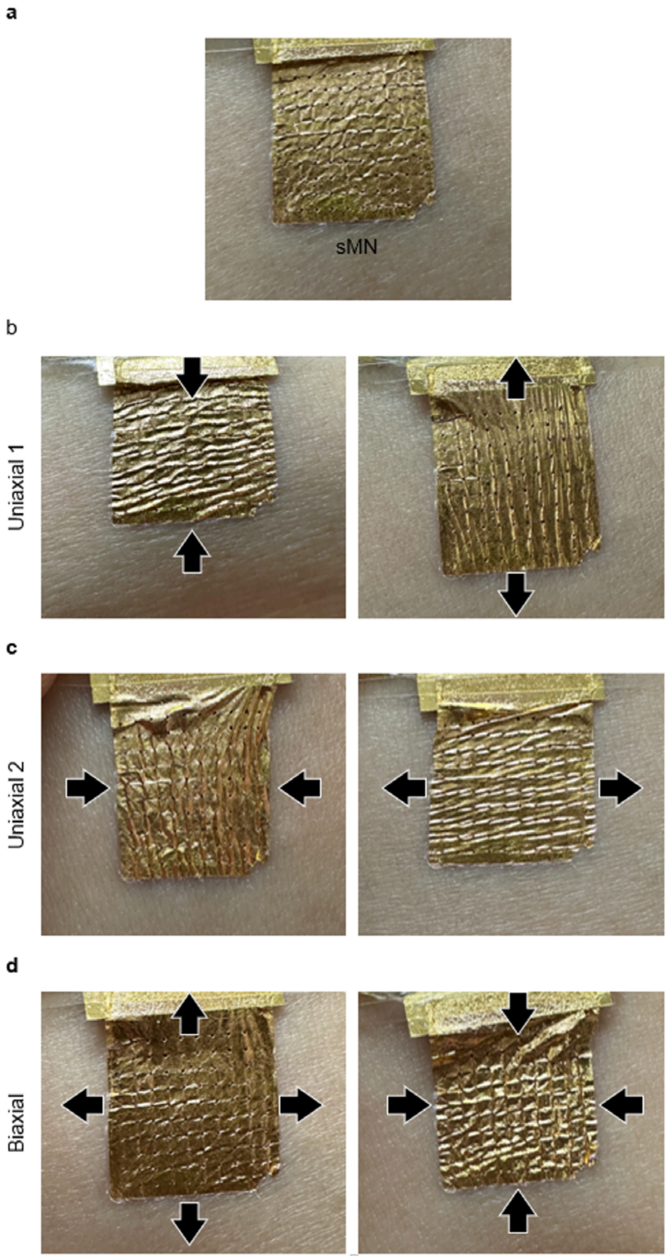

Supplementary Fig. 16 | Mechanical adaptability of the sMN electrode on human skin under uniaxial and biaxial deformation. a, sMN electrode attached to the human forearm skin. b-c, Optical images of the sMN under uniaxial compression and stretching, (**b**) top-bottom and (**c**) left-right, showing reversible wrinkling and conformal adhesion. **d**, Biaxial stretching and compression, demonstrating uniform mechanical adaptability and structural integrity under multidirectional strain.

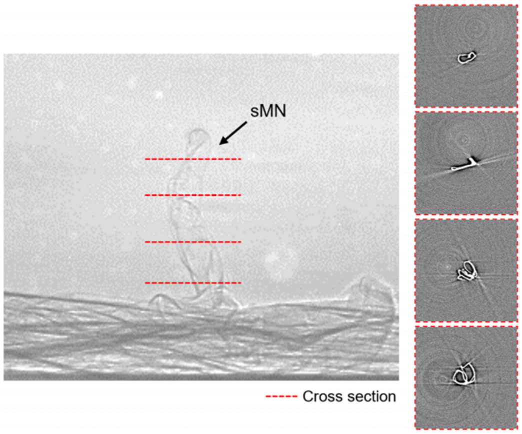

Supplementary Fig. 17 | Structural integrity of sMN after repeated biaxial deformation. sMN patches were applied to human skin and subjected to 100 cycles of biaxial compression–stretching to simulate realistic skin motion. Synchrotron micro-CT imaging confirmed that the microneedle structures maintained their integrity without tearing or wearing. The left panel shows a side view of an intact sMN post-test, while the right panels display cross-sectional views at different heights (red dashed lines), demonstrating that the sMN remained structurally stable and continuous across all layers.

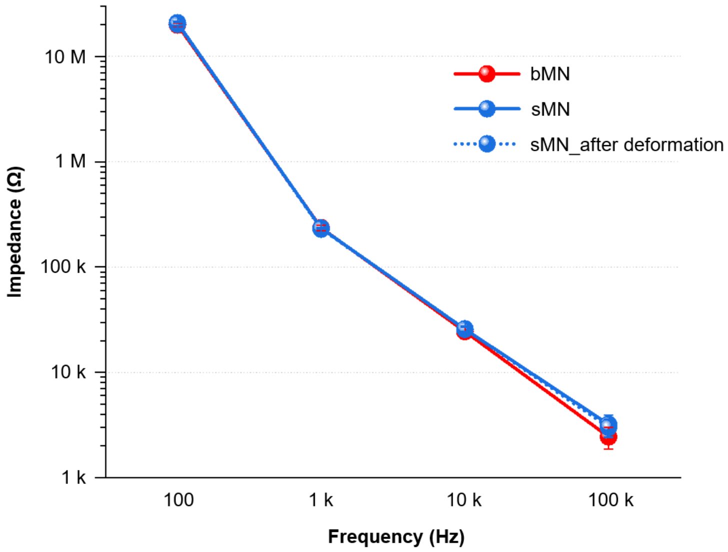

Supplementary Fig. 18 | Impedance spectra of bMN and sMN before and after deformation. Frequency-dependent impedance (100 Hz to 100 kHz) of bMN (red) and sMN (blue) electrodes, compared with sMN after repeated deformation (blue dotted line). The sMN maintains stable impedance characteristics across all measured frequencies even after mechanical deformation, indicating excellent electrochemical stability and mechanical robustness.

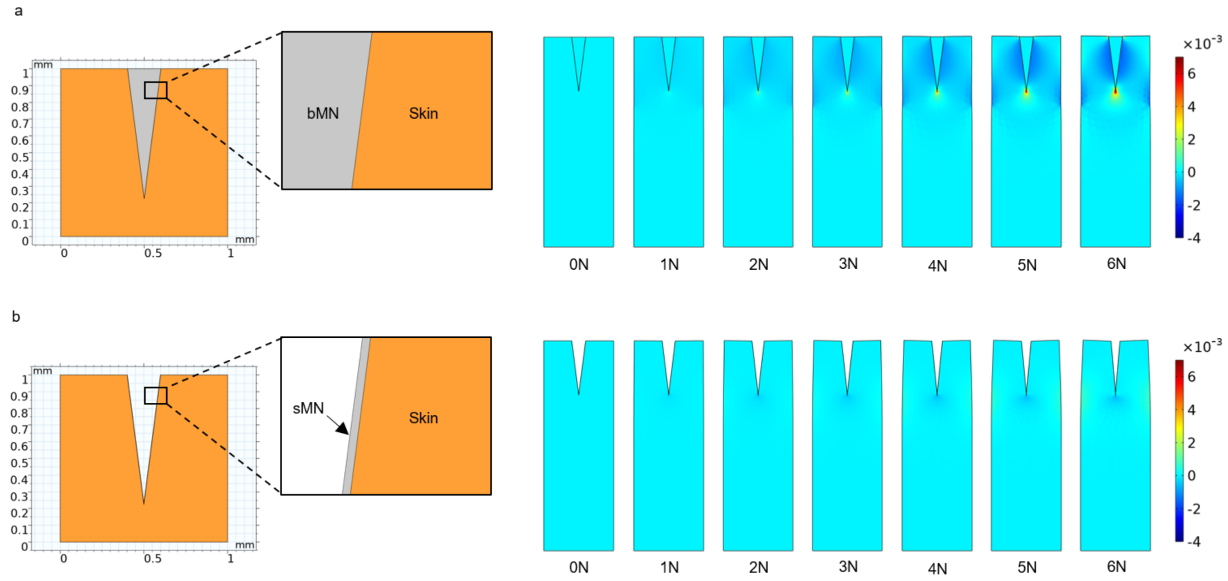

Supplementary Fig. 19 | Finite element analysis of volumetric strain distribution for bMN and sMN under lateral loading. a, Simulation of bMN insertion into skin, showing volumetric strain distribution as lateral forces (0–6 N) are applied. The rigid structure of the bMN concentrates stress near the insertion site, resulting in localized strain amplification (highlighted in red). **b**, Simulation of sMN under identical loading conditions. The soft, thin-walled structure of the sMN dissipates stress more evenly, leading to reduced and broadly distributed strain fields within the surrounding tissue. Color scale represents volumetric strain ($\times 10^{-3}$).

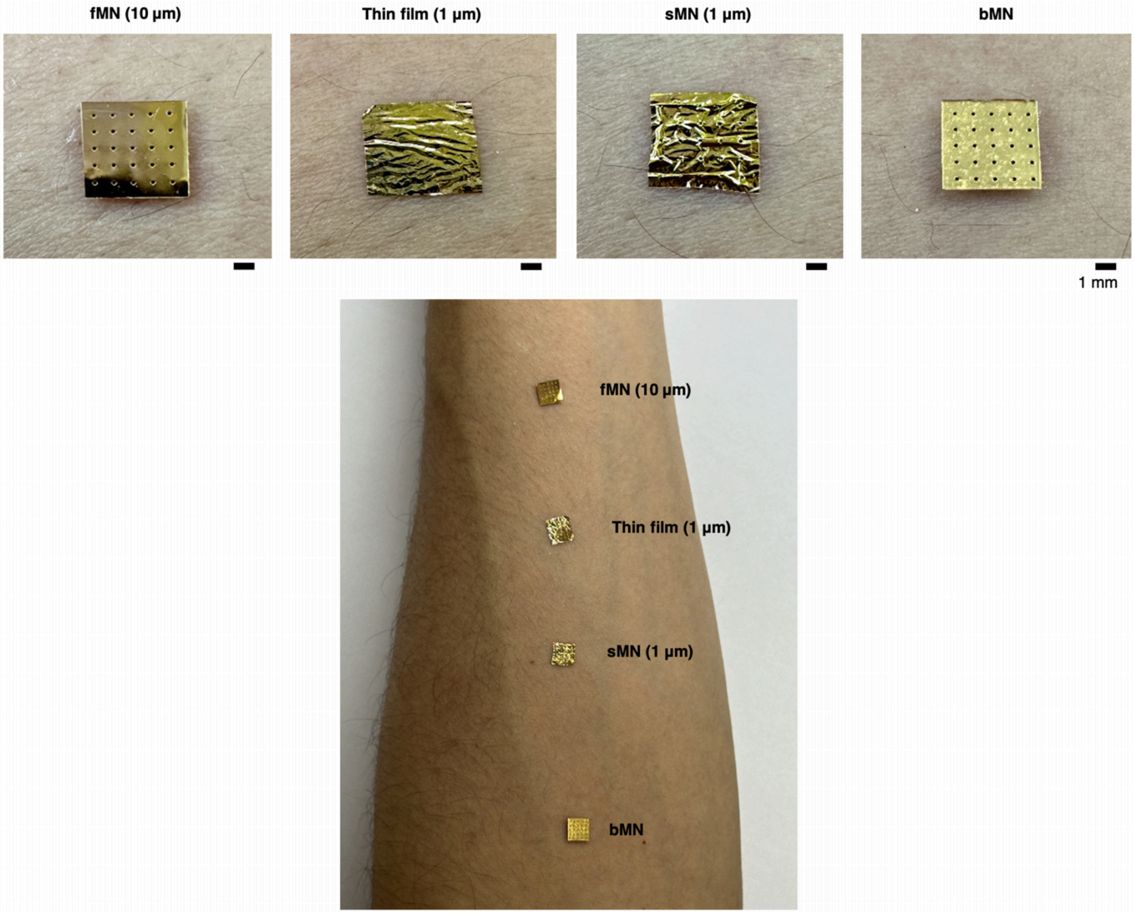

Supplementary Fig. 20 | Psychological evaluation setup for on-skin devices. A fMN, a thin film, an sMN patch, and a bMN patch were randomly applied to the forearms of human volunteers.

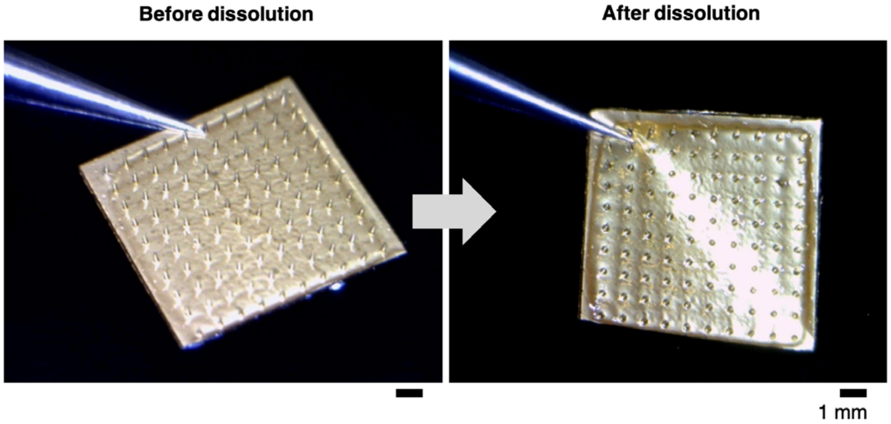

Supplementary Fig. 21 | Free-standing fMN electrode. An fMN patch features stiff and free-standing characteristics even after the dissolution of the effervescent sacrificial substrate.

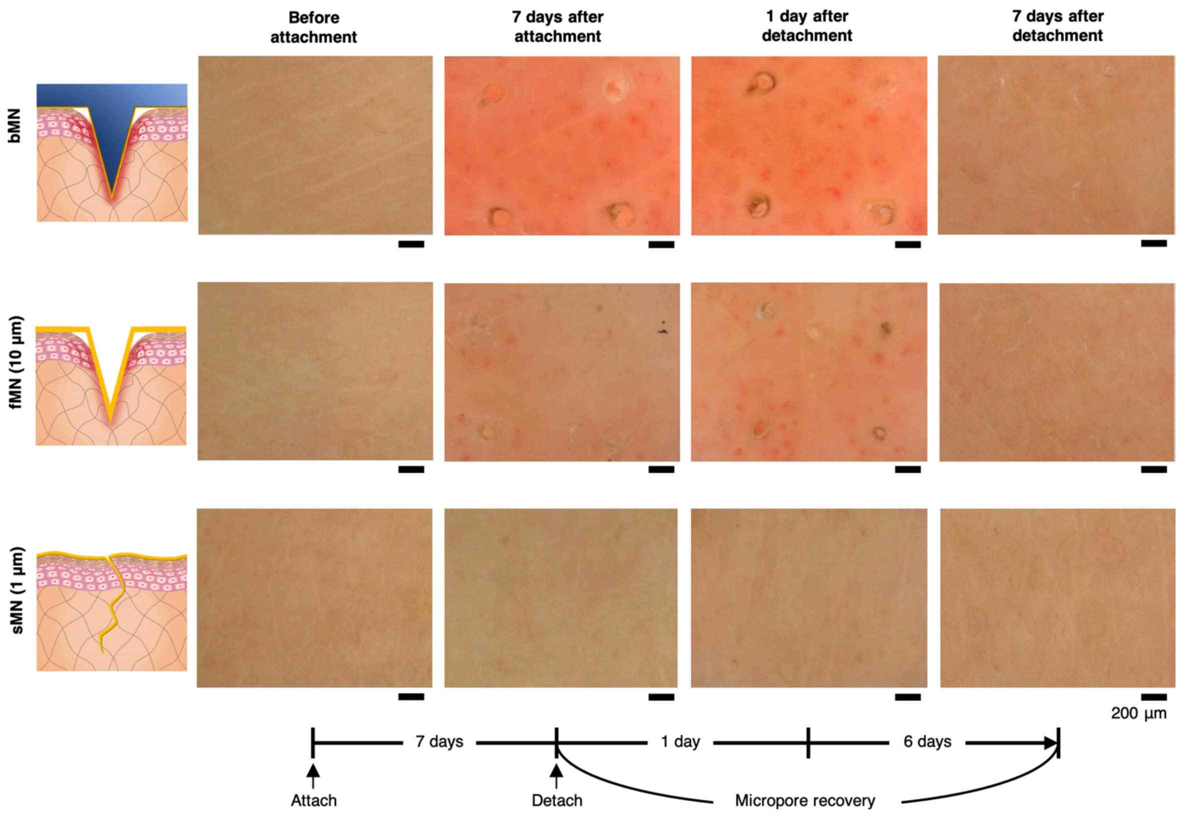

Supplementary Fig. 22 | Long-term monitoring of redness caused by different types of MN patches. Skin redness caused by the application of on-skin devices was evaluated using a hand-held digital microscope. Consistent lighting conditions were maintained throughout the experiments. bMN, fMN, and sMN patches were attached to the skin for 7 days, and the skin was monitored after removal, as well as 1 day and 7 days post-removal, to compare with the baseline skin condition before device application.

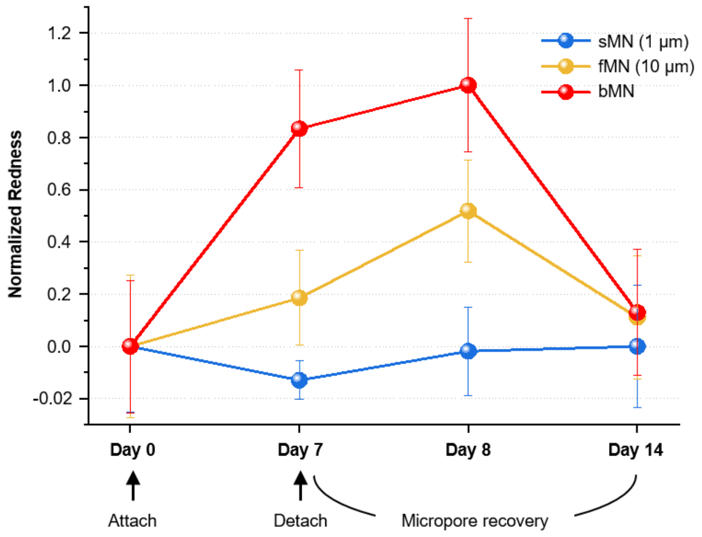

Supplementary Fig. 23 | Skin redness quantification for the long-term use of different types of MN patches. While redness caused by the bMN increased significantly by 23.68% and 28.42% on days 7 and 8, respectively, the fMN showed a relatively lower but still noticeable increase of 5.29% and 14.81% on the same days. Notably, sMN showed no measurable change in redness throughout the entire observation period. In all cases, redness was alleviated 14 days after device removal.

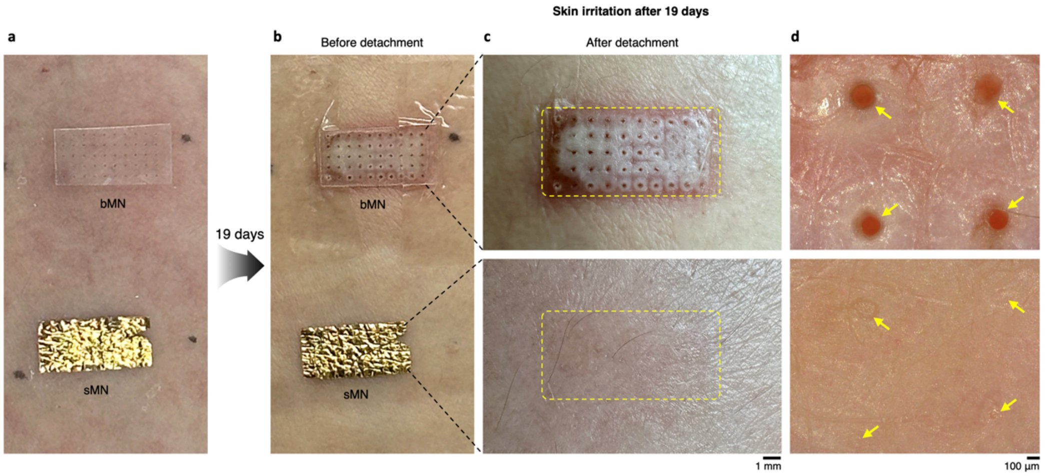

Supplementary Fig. 24 | Long-term *in vivo* assessment of skin responses to MN electrodes over 19 days. a, Representative images of bMN (top) and sMN (bottom) patches immediately after application on human forearm skin. **b**, Macroscopic appearance of the patches after 19 days of continuous application, prior to detachment. **c**, Skin sites immediately after detachment on day 19, with yellow dashed boxes marking the patch areas. The bMN site exhibited persistent erythema and punctate erosions, whereas the sMN site showed only mild, transient erythema without barrier disruption. **d**, Magnified optical microscopy images of the corresponding sites. Yellow arrows indicate insertion marks caused by microneedles. The bMN induced punctate erosions with focal epidermal necrosis, while the sMN left only faint and reversible puncture marks.

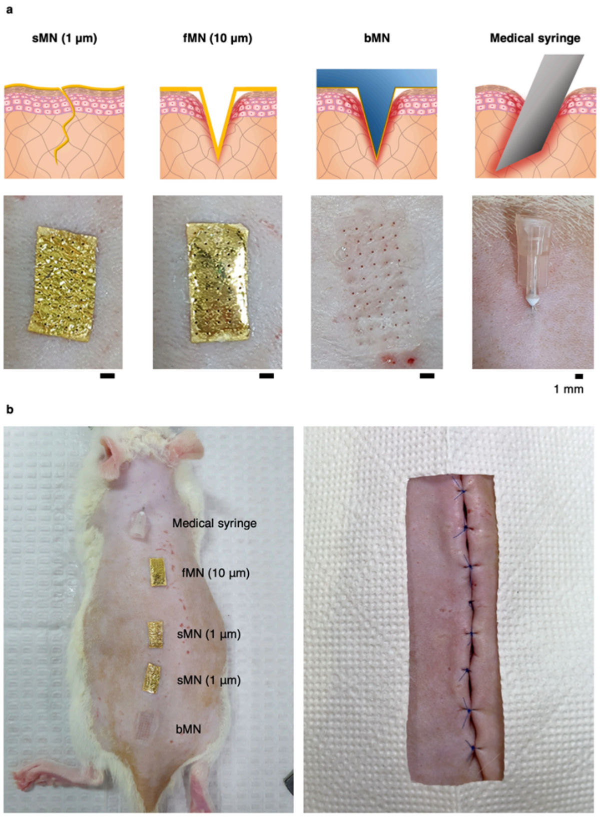

Supplementary Fig. 25 | Animal experiment for *in vivo* histological analysis. a, sMN, fMN, bMN, and a medical syringe (26-gauge) were inserted into the SD rat skin *in vivo* for histological analysis. The dorsal area of the rat was shaved with wax prior to the experiment. **b**, The location of each device was randomly distributed on the skin (left). After insertion, the skin was stitched to ensure that the devices remained in position (right).

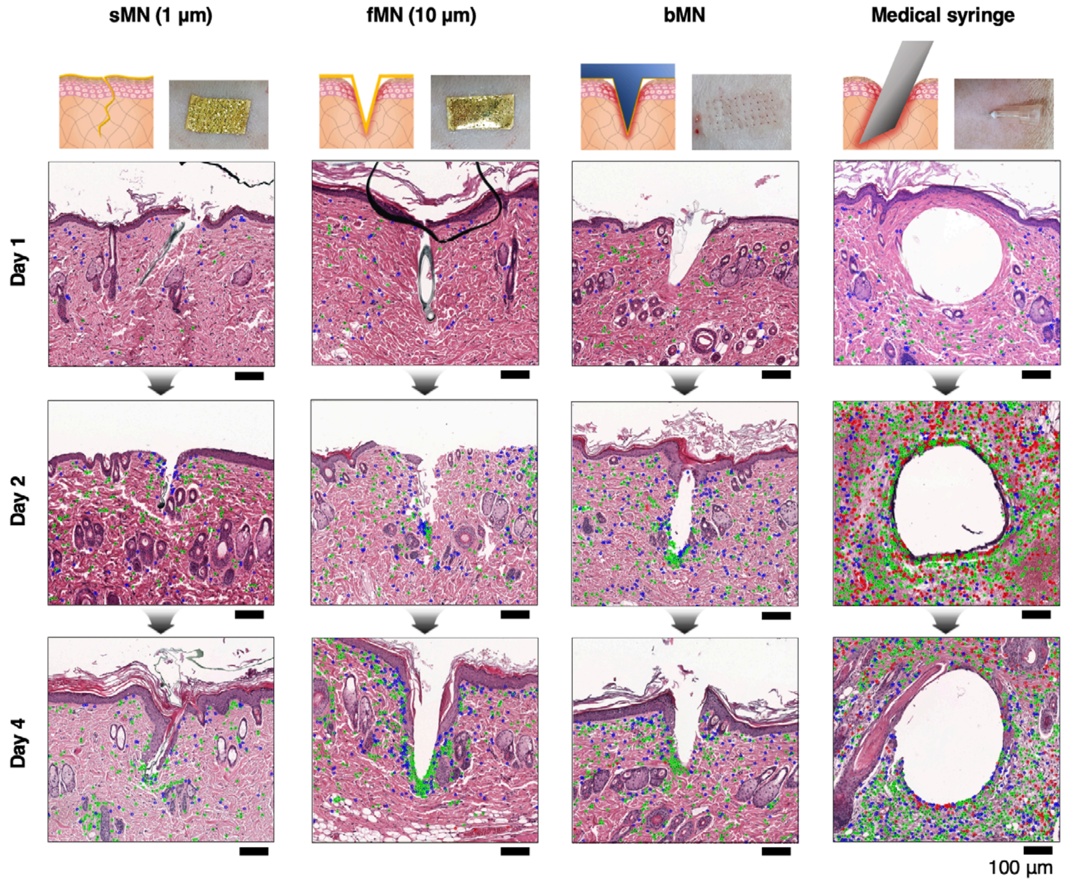

Supplementary Fig. 26 | Color-marked inflammatory cells in a histological image of SD rat skin showing the trace of inserted sMN, fMN, bMN, and a medical syringe. Three representative types of inflammatory cells were marked with different colors by the pathologist: macrophages in green, lymphocytes in blue, and neutrophils in red.

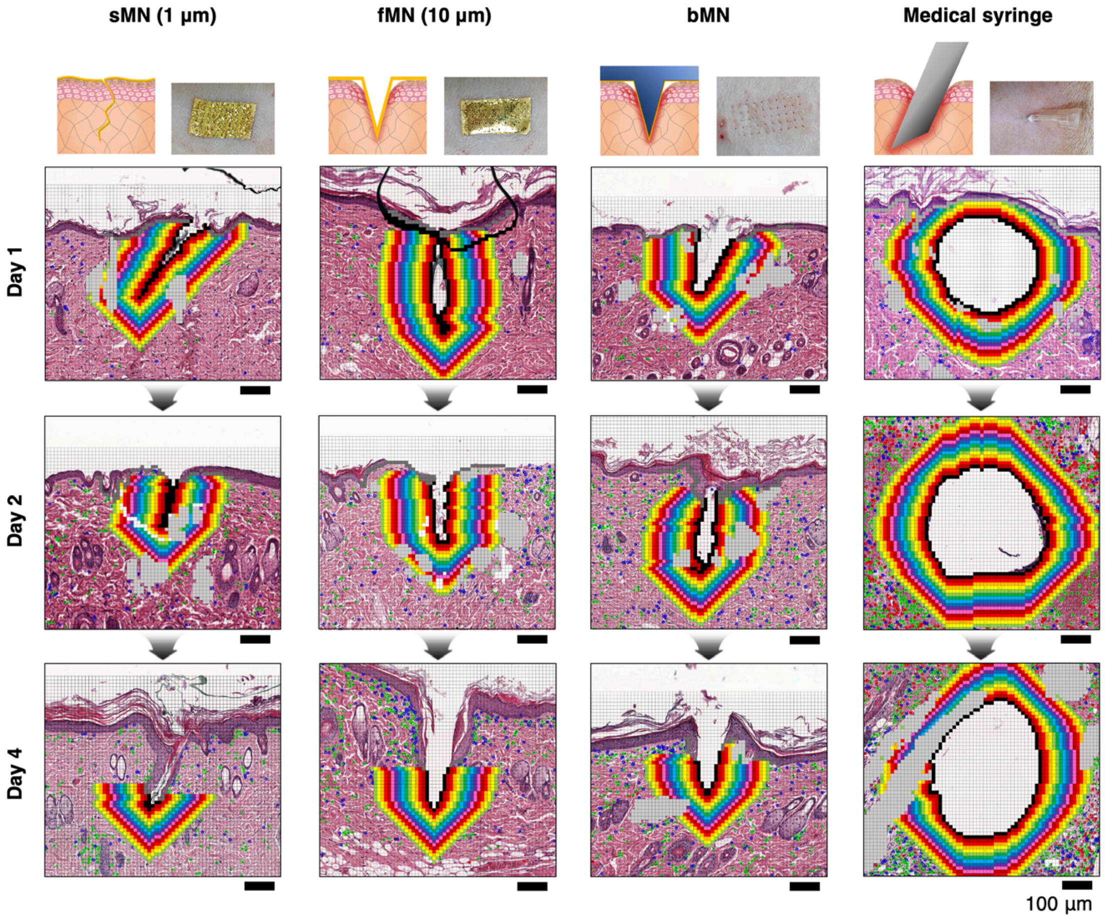

Supplementary Fig. 27 | Lattice-assisted inflammatory cell counting and analysis of their distribution. A virtual lattice is overlaid onto histological images to calculate the density of inflammatory cells. Each unit cell is colored based on its distance from the surface of the inserted device.

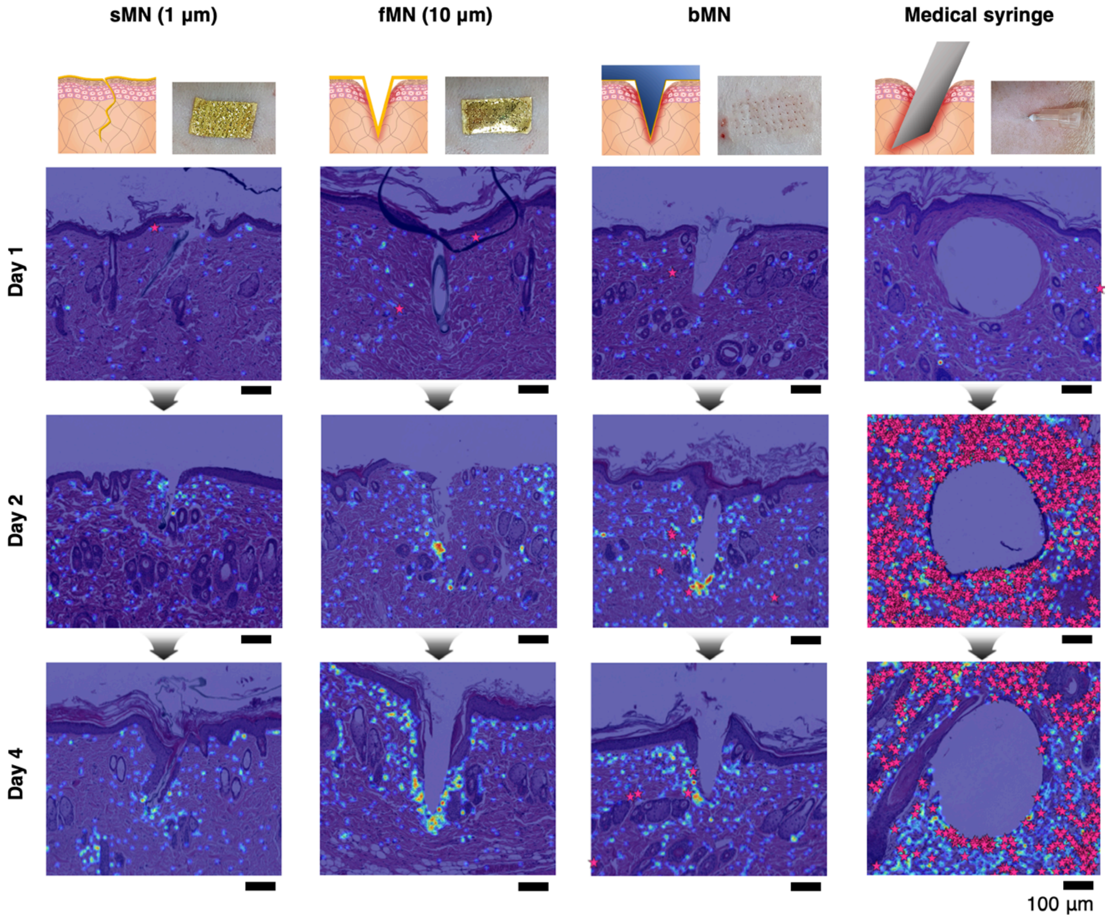

Supplementary Fig. 28 | Histological analysis on SD rat showing neutrophil. Neutrophil was marked with a pink-colored star-shaped indicator. Neutrophil clearly appears in the histological images of bMN and medical syringe from day 2.

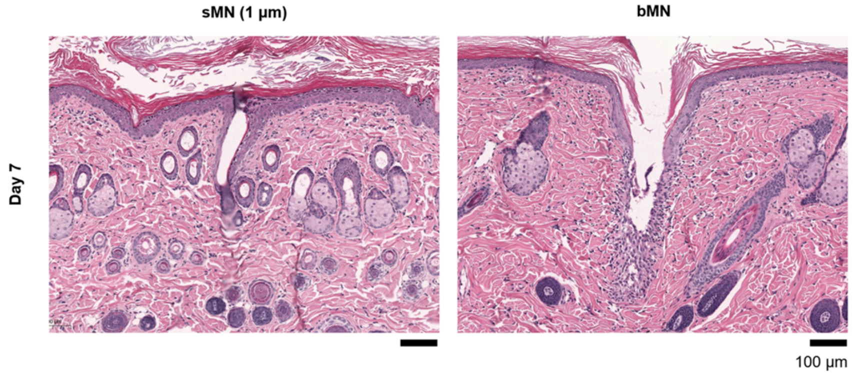

Supplementary Fig. 29 | Histological evaluation of skin tissue at day 7 post-insertion. sMN (left) and bMN (right) insertion sites in SD rat skin, stained with hematoxylin and eosin (H&E). The sMN group shows minimal residual tissue disruption and well-preserved epidermal/dermal structures, whereas the bMN group exhibits persistent small tissue damages and localized inflammatory cell infiltration.

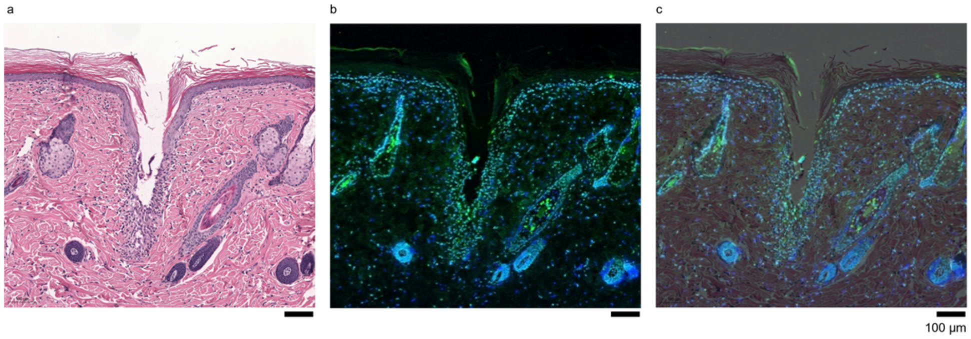

Supplementary Fig. 30 | Validation of histological interpretation by TUNEL assay for bMN at day 7. a, Hematoxylin and eosin (H&E) staining of SD rat skin at the bMN insertion site, showing localized tissue disruption and inflammatory cell infiltration. **b**, TUNEL assay indicating apoptotic cells (green) in the same region, corresponding well to areas of tissue damage observed in H&E staining. **c**, Merged image of H&E and TUNEL results, demonstrating consistency between histological evaluation and apoptotic marker localization.

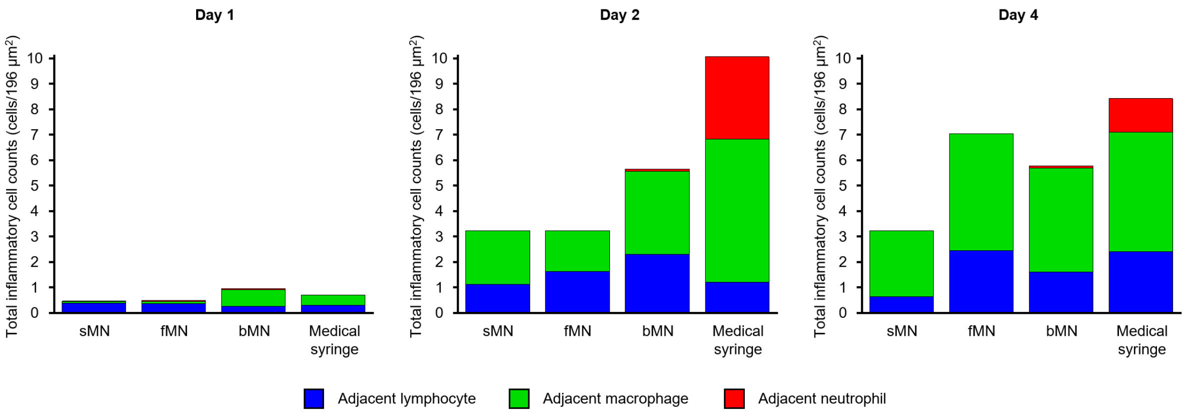

Supplementary Fig. 31 | Immune cell-type quantification around different insertion devices over time. Stacked bar plots show the total counts and distribution of lymphocytes (blue), macrophages (green), and neutrophils (red) within a 196 μm² area adjacent to the insertion site for sMN, fMN, bMN, and a 26G medical syringe at Day 1, 2, and 4. sMN shows minimal immune cell infiltration and rapid transition to macrophage-dominant profiles, whereas bMN and syringe induce stronger neutrophil-driven acute inflammation that persists over time.

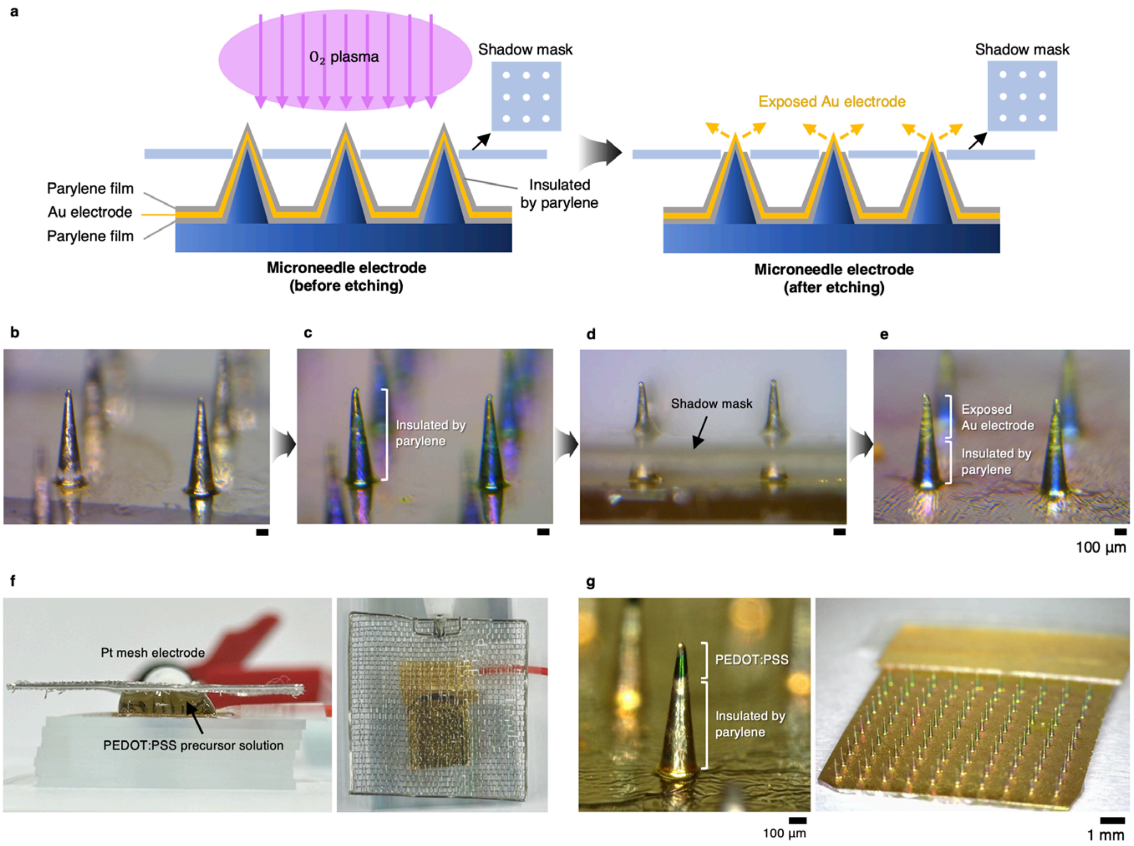

Supplementary Fig. 32 | Selective etching and coating on the tip of MN electrodes. a, Schematic illustration of reactive ion etching (RIE) using a shadow mask, selectively etching the insulation layers at the tip of MN electrodes. Optical images showing the stepwise fabrication process: **b**, effervescent sacrificial MN electrodes were first coated with parylene thin film followed by gold deposition; **c**, the MN electrodes were then insulated with an additional parylene thin film layer; **d**, a shadow mask was applied to expose the tips of MN electrodes; and **e**, RIE was used to selectively etch the exposed tips. The resulting color difference—yellow (etched) at the tip and blue (insulated) at the base—indicates successful selective etching. **f**, Experimental setup for the electrochemical deposition of PEDOT:PSS onto the exposed electrode tips using a Pt-mesh counter electrode (left) and its top view (right). **g**, Final MN electrodes after deposition, showing PEDOT:PSS coating localized at the tip (left) and the completed electrode array (right).

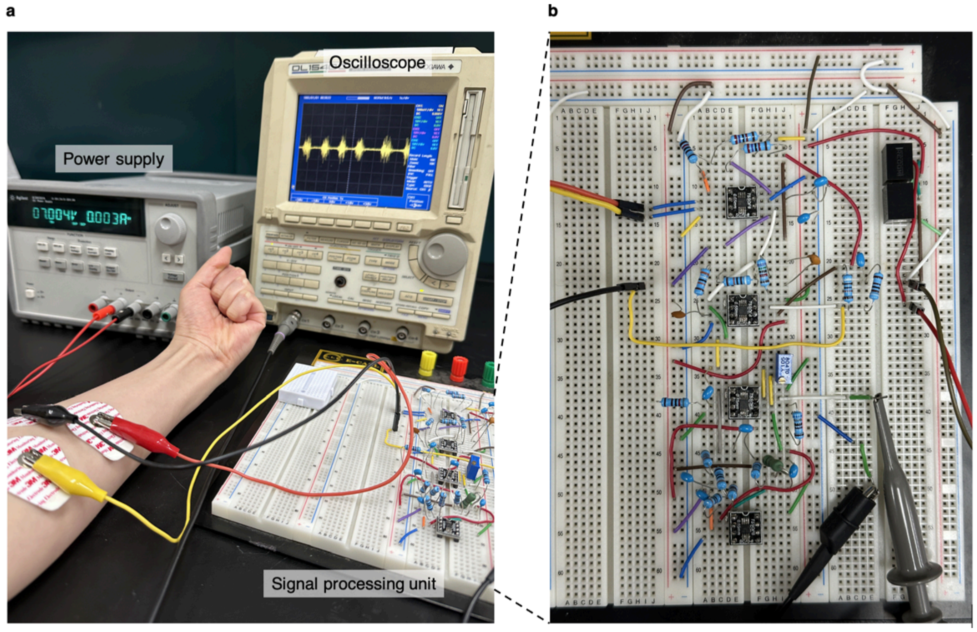

Supplementary Fig. 33 | Setup for a customized EMG measurement system. a, Experimental setup showing the arrangement of an oscilloscope, power supply, and breadboard with a signal processing unit. **b**, Circuit configuration on the breadboard for EMG measurement.

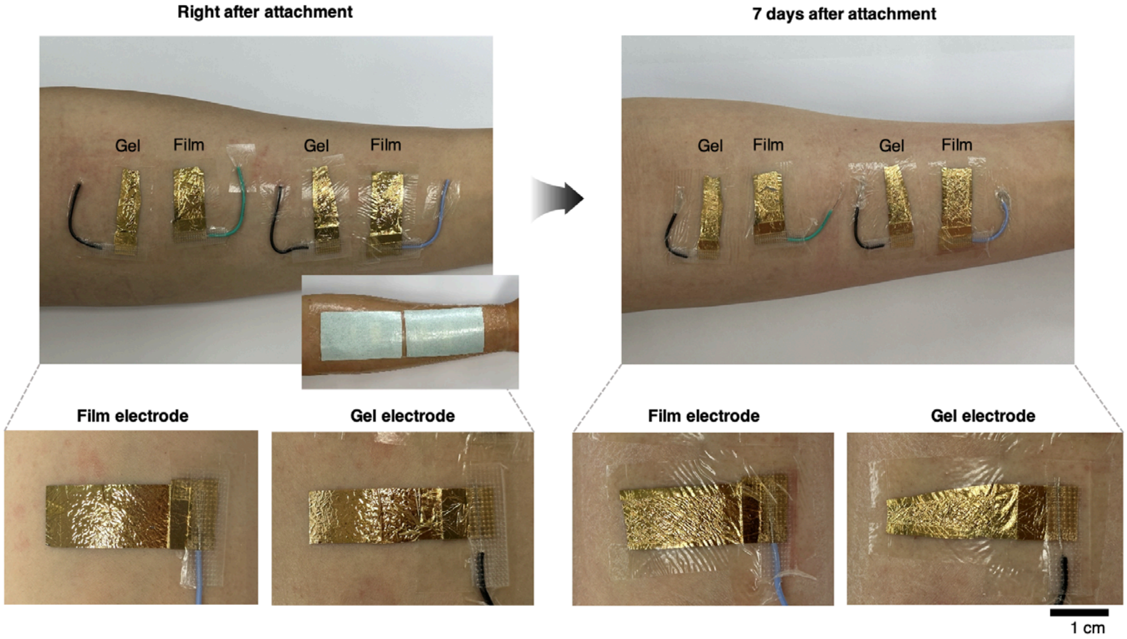

Supplementary Fig. 34 | Long-term acquisition of EMG using gel- and film-type bioelectrodes on human forearm. Gel- and film-type bioelectrodes were attached to the forearm of a human volunteer. The electrodes were covered with soft fabric to protect them from unexpected external disturbances. The attachment of each device was confirmed after 7 days.

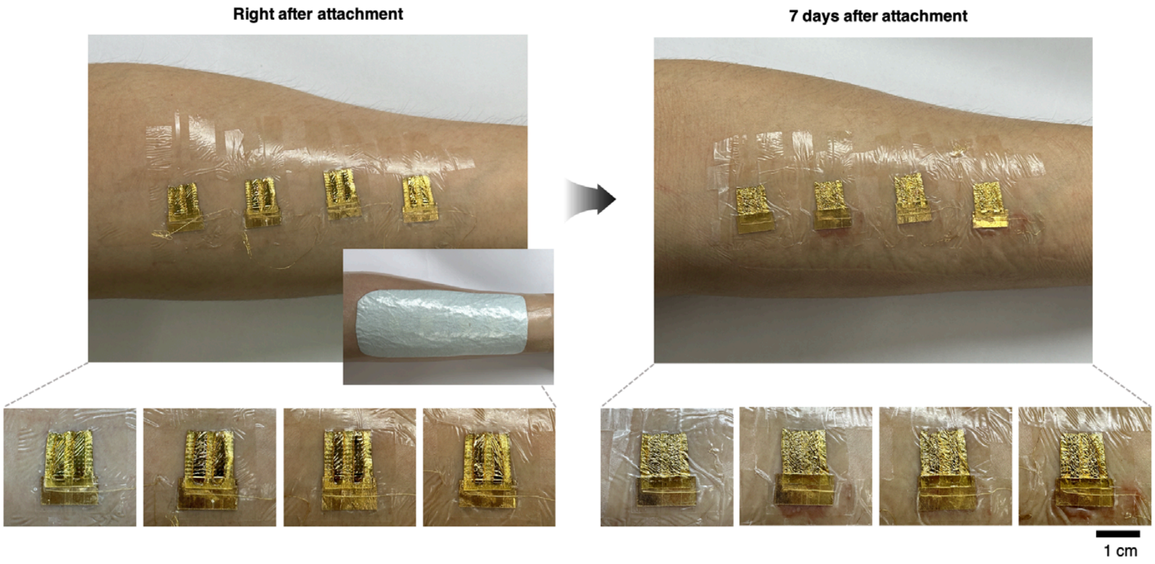

Supplementary Fig. 35 | Long-term acquisition of EMG using sMN bioelectrodes on human forearm. sMN bioelectrodes were attached to the forearm of a human volunteer. The electrodes were covered with soft fabric to protect them from unexpected external disturbances. The attachment of each device was confirmed after 7 days.

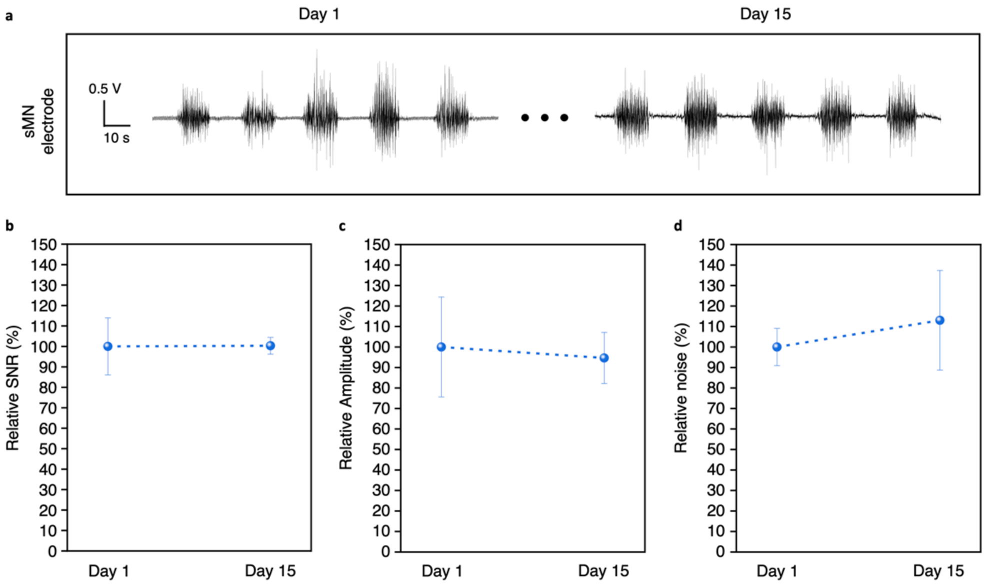

Supplementary Fig. 36 | Long-term electrophysiological stability of sMN electrodes. Electromyography (EMG) signals recorded from a human subject using sMN electrodes over 15 consecutive days. The signal-to-noise ratio (SNR) remained stable throughout the recording period, demonstrating reproducible and sustained electrophysiological monitoring performance. Data are presented as mean ± SD, confirming negligible performance degradation during extended application.

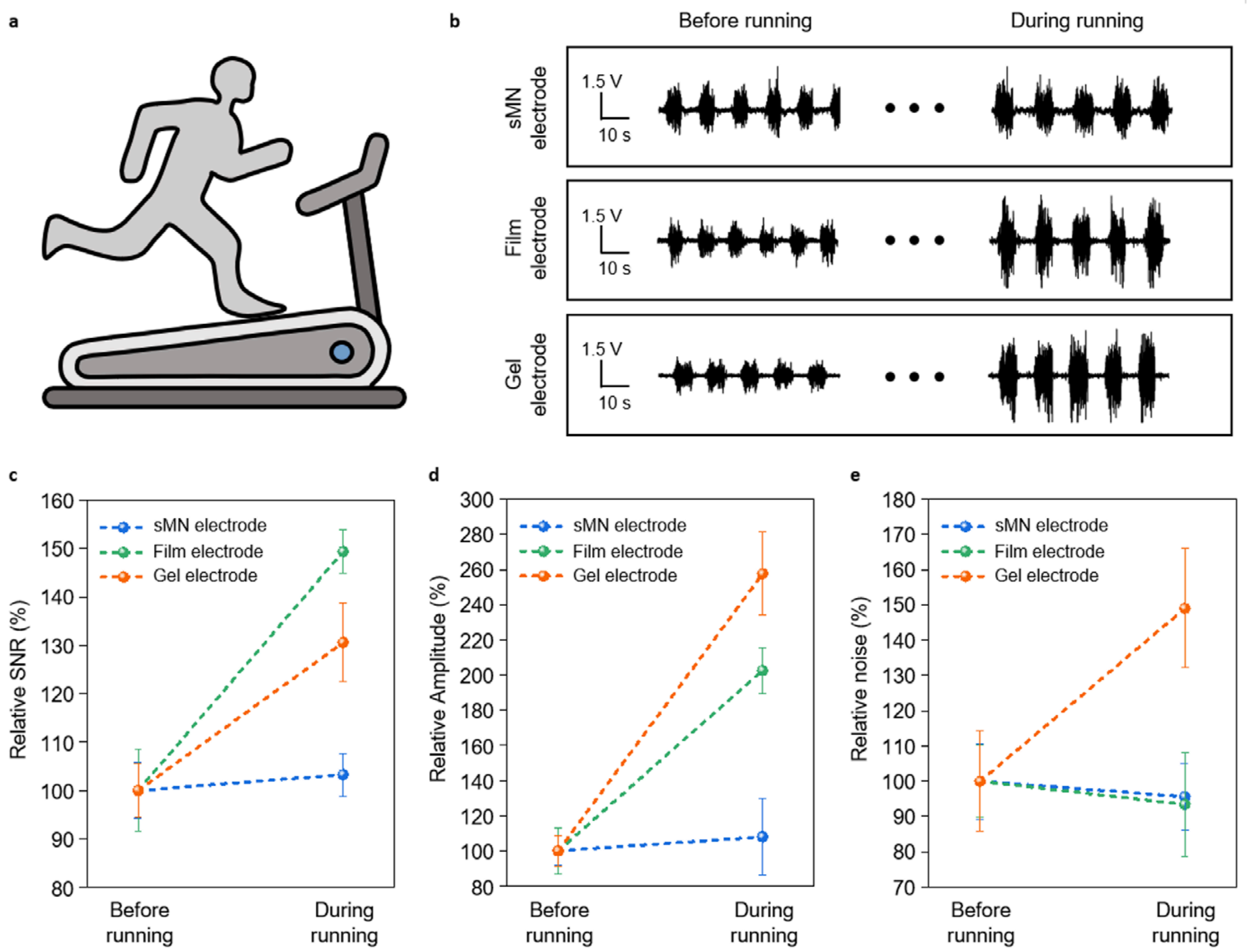

Supplementary Fig. 37 | Electromyography (EMG) performance of electrodes under treadmill running and sweating conditions. a, Schematic illustration of the treadmill-running test setup used to evaluate electrode stability under dynamic motion and perspiration. **b**, EMG signals recorded with sMN, film, and gel electrodes before and during running. The sMN maintained consistent signal morphology, whereas the film and gel electrodes exhibited unintended amplitude fluctuations due to motion and sweat. **c–e**, Quantitative comparison of relative signal-to-noise ratio (**c**), amplitude (**d**), and noise (**e**) before and during running for each electrode type. While no baseline drift was observed in any group, the sMN showed superior signal stability compared to film and gel electrodes.

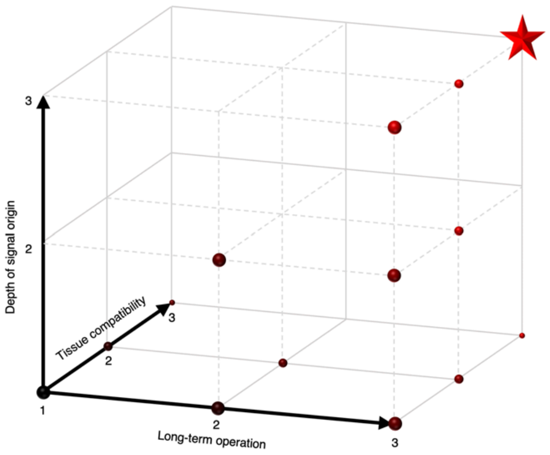

Supplementary Fig. 38 | Three-dimensional comparative analysis of wearable bioelectronic devices. Representative devices were mapped into the three-axis framework (X: tissue compatibility, Y: long-term operation, Z: depth of signal origin). The red star highlights the position of the proposed sMN system, which uniquely combines high tissue compliance, long-term intrinsic conductivity, and selective percutaneous signal acquisition. Other points correspond to representative studies, as detailed in Supplementary Table S5 and S6. The plot demonstrates the superior balance of mechanical adaptability, stability, and signal fidelity achieved in this work.

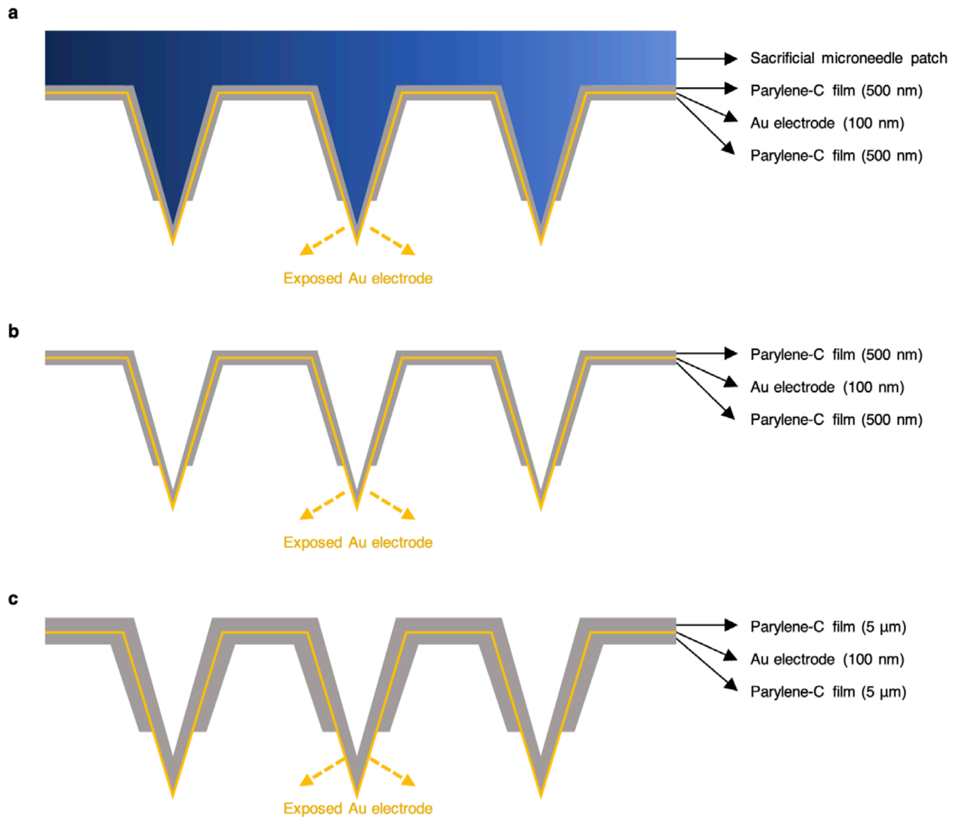

Supplementary Fig. 39 | Structure of bMN, sMN, and fMN. a, bMN consists of a PVP–citric acid–sodium bicarbonate core coated with a parylene–gold–parylene trilayer (500 nm/100 nm/500 nm). **b**, After core dissolution, only the ultrathin parylene–gold–parylene film remains, forming the flexible sMN. **c**, fMN features a thicker parylene base layer (5 μm/100 nm/5 μm), resulting in stiffness intermediate between bMN and sMN.

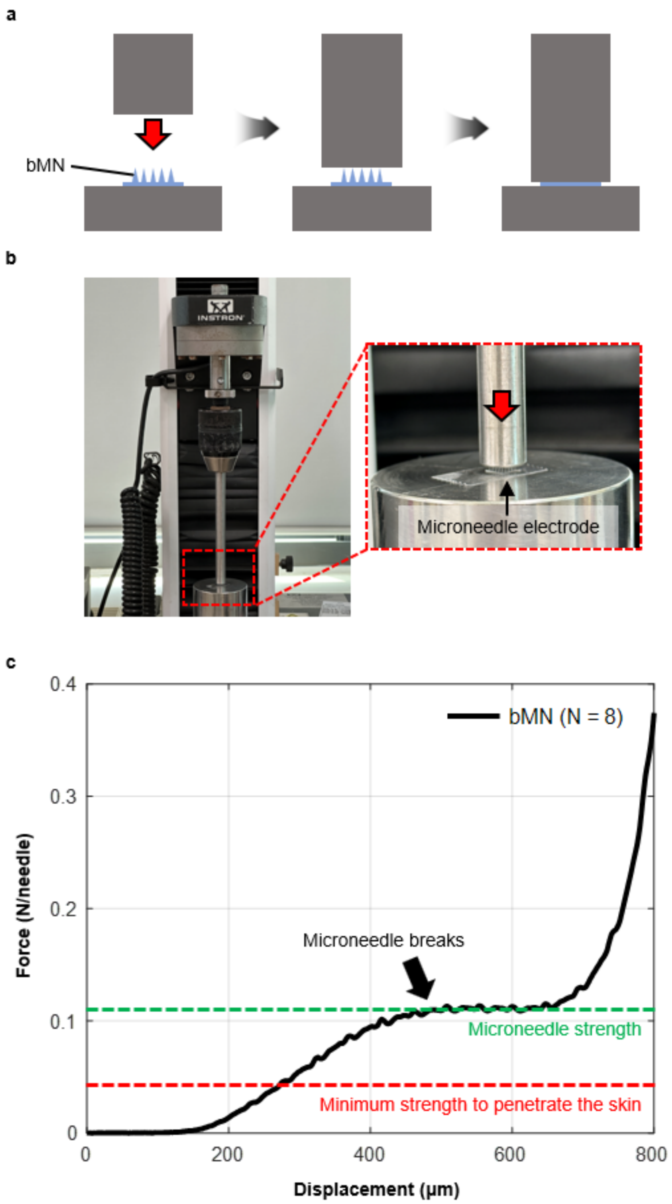

Supplementary Fig. 40 | Measurement of the fracture force of the MN electrodes. A bMN patch was subjected to the uniaxial compression to measure the fracture force of the bMNs. **a**, Schematic of the bMN patch tested by uniaxial compression machine and **b**, its experimental setup. **c**, Inflection point of the graph indicates the average fracture force of 0.11N/needle which is higher than the required minimal strength (0.05N/needle) for b MN to penetrate the stratum corneum of the skin.

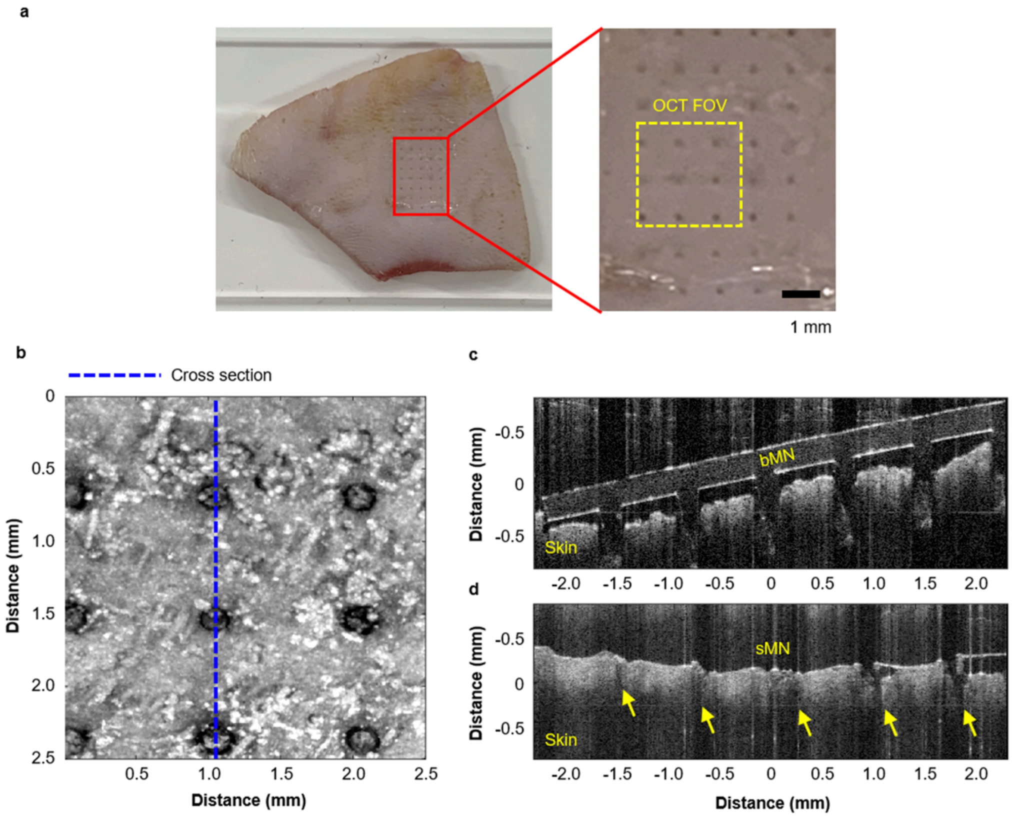

Supplementary Fig. 41 | Visualization of the MN insertion into SD rat skin using optical coherence tomography (OCT). The insertion depth and profile of the microneedles were analyzed via optical coherence tomography (OCT). **a**, MN patch applied onto ex vivo SD rat skin. **b**, Top-view OCT image of the insertion site. **c**, Cross-sectional OCT image of the bMN showing the insertion profile. **d**, Cross-sectional OCT image of the sMN, with yellow arrows indicating the outline of the soft microneedle tips.

	Soft MN (This Work)	Rigid MN	Rigid MN with thin-film patches	Rigid MN with stretchable patch
Pre-insertion modulus	High (GPa-scale)	High (GPa-scale)	High (GPa-scale)	High (GPa-scale)
Post-insertion modulus	Low (~100 kPa)	High (GPa-scale)	High (GPa-scale)	High (GPa-scale)
Physical decoupling	O	O	O	O
Conformality	O	X	O	O
Long-term signal stability	O	-	-	-
Cell-stress-free integration	O	X	X	X

Supplementary Table 1 | Comparative analysis of microneedle-based bioelectrodes. This table compares the soft microneedle (sMN) bioelectrode developed in this work with existing classes of microneedle (MN)-based electrodes, including rigid MNs, MNs integrated with thin-film patches, and MNs coupled with stretchable platforms. Parameters include pre- and post-insertion modulus, physical decoupling, conformability, long-term signal stability, and cell-stress-free integration. The sMN platform uniquely exhibits post-insertion softening, stable bioelectrical interfacing, and chronic inflammation resilience.

	p-value
bMN vs fMN	*
bMN vs sMN	***
bMN vs sMN	*

Supplementary Table 2 | Statistical significance of mechanical performance among MN types. One-way ANOVA followed by post-hoc Tukey's multiple comparison test was performed to compare bMN, fMN, and sMN groups. Significance levels are indicated as *$p < 0.05$, **p<0.01, ***p<0.01.

	Film	sMN	fMN	bMN
Film	-	**	***	***
sMN	**	-	***	***
fMN	***	***	-	*
bMN	***	***	*	-

Supplementary Table 3 | Statistical significance of mechanical performance among MN types. One-way ANOVA followed by post-hoc Tukey's multiple comparison test was performed to compare bMN, fMN, and sMN groups. Significance levels are indicated as *$p < 0.05$, **p<0.01, ***p<0.01.

	sMN (This Work)	Gel electrode	Film electrode	Invasive electrode
Signal quality	5	2	2	5
Biocompatibility	4	2 (irritation)	5	1 (inflammation)
User comfort	4	2 (wet, sticky)	4	1
Conformality	5	2 (thick)	4 (stiff)	N/A
Long-term usability	4	1 (gel dry-out)	2 (adhesion loss)	1 (blood-fouling)
Fabrication complexity	2	3	3	4

Supplementary Table 4 | Comparison of sMN with gel, film, and invasive electrodes across key performance metrics. Summary of performance characteristics for sMN (this work), gel electrodes, film electrodes, and invasive electrodes across six key evaluation criteria: signal quality, biocompatibility, user comfort, conformality, long-term usability, and fabrication complexity. Scores are rated on a scale of 1 to 5, based on experimental observations and literature references. Parentheses indicate the primary limitations associated with each electrode type. Score definition: 5 = excellent, 4 = good, 3 = moderate, 2 = poor, 1 = very poor

	Level		
Axis	1	2	3
X<br>(Tissue compatibility)	Rigid/Liquid mismatch	Moderately compliant<br>(Flexible/thin)	Highly compliant<br>(Soft/porous/stretchable)
Y<br>(Long-term operation)	Temporal conductivity<br>(Ionic electrolyte)	Sustained conductivity<br>(Ionic electrolyte+Humectant)	Intrinsic conductivity<br>(Solid-state electrolyte)
Z<br>(Depth of signal origin)	Epidermal<br>(Superficial)	Percutaneous<br>(Non-passivated)	Percutaneous<br>(Passivated+Selective)

Supplementary Table 5 | Classification framework for evaluating wearable bioelectronic interfaces. This table summarizes the three quantitative axes used to evaluate different classes of wearable bioelectronic devices: (X) tissue compatibility, (Y) long-term operation, and (Z) depth of signal origin. Each axis is subdivided into three levels based on structural, material, and functional criteria. This framework allows a standardized and quantitative comparison of various reported devices. This tabular form directly maps to the 3D comparison chart (Supplementary Fig. 38) and provides the detailed basis for plotting.

X (Tissue compatibility)	Y (Long-term operation)	Z (Depth of signal origin)	Reference list
1	1	1	11, S1-S9, 31
1	1	2	
1	1	3	
1	2	1	S10-S12
1	2	2	S13
1	2	3	
1	3	1	S14-S15
1	3	2	S16-S18
1	3	3	S19-S22
2	1	1	S23-S26
2	1	2	
2	1	3	
2	2	1	S27
2	2	2	
2	2	3	
2	3	1	S28-S29
2	3	2	S30-S34
2	3	3	27, 32, S35, 26
3	1	1	S36-S40
3	1	2	
3	1	3	
3	2	1	
3	2	2	
3	2	3	
3	3	1	7, S41, 1, 8, 21, S42, 25, S43-S47
3	3	2	
3	3	3	This work (sMN)

Supplementary Table 6 | Device classification across X–Y–Z axes based on representative literature. Representative devices reported in the literature were classified into the three-axis framework (X: tissue compatibility, Y: long-term operation, Z: depth of signal origin). Each entry specifies the corresponding device category and reference papers. This tabular form directly maps to the 3D comparison chart (Supplementary Fig. 38) and provides the detailed basis for plotting.

Supplementary Videos

Supplementary Video 1 | Rapid removal of effervescent sacrificial substrate.

Addition of water effectively removes the sacrificial materials not only from the substrate but also inside the micro-cavities at the tip of MNs.

Supplementary Video 2 | Mechanical durability test for sMN array by repetitive extreme deformation.

The resilience of sMNs lies in their ability to relieve mechanical stress by flexibly adapting their shape without resisting the external force.

Supplementary Video 3 | 3D rendering of the X-ray images of the sMN electrode inserted into the rat skin *ex vivo.*

Synchrotron X-ray micro CT imaging reveals highly flexible sMN is significantly deformed within the skin, conforming closely to the internal tissue structures without causing mechanical stress or damage.

Supplementary Video 4 | Detachment of sMN from the human skin.

When the sMN patch is peeled off, the sMNs are smoothly withdrawn from the skin without breakage of damage.

Supplementary Video 5 | The integrated circuit demonstrated involuntary motion-insensitive system – Voluntary action.

Stable electromyographic signals, recorded from the sMN applied to the forearm, clearly reflect repeated voluntary hand clenching and releasing motions.

Supplementary Video 6 | The integrated circuit demonstrated involuntary motion-insensitive system – Involuntary action (wrist bending).

No significant electromyographic signals are detected from the sMN applied to the forearm during repeated passive wrist flexion and extension.

Supplementary Video 7 | The integrated circuit demonstrated involuntary motion-insensitive system – Involuntary action (forearm lifting).

No significant electromyographic signals are detected from the sMN applied to the forearm during repeated passive forearm lifting.

Supplementary Video 8 | Real-time data transfer of EMG signal on the human skin using a commercially available signal processing system.

EMG signals acquired from the sMN electrode are transmitted in real time and processed using a commercial signal processing system.

Supplementary Video 9 | Real-time EMG recording during dynamic motion and sweating.
sMN, film, and gel electrodes were attached to the human forearm (extensor muscles) for electromyography (EMG) acquisition. The subject performed treadmill running to induce dynamic motion and perspiration, enabling direct comparison of signal quality, motion artifacts,

and hydration-induced fluctuations among the three electrode types. The dynamic treadmill running demonstrates that the sMN electrode maintained stable and noise-resistant EMG signals, while film and gel electrodes exhibited signal distortion and amplitude variability under motion and sweat.

Supporting references

S1. Miyamoto, Akihito, et al. "Inflammation-free, gas-permeable, lightweight, stretchable on-skin electronics with nanomeshes." *Nature nanotechnology* 12.9 (2017): 907-913.

S2. Lee, Sunghoon, et al. "Nanomesh pressure sensor for monitoring finger manipulation without sensory interference." *Science* 370.6519 (2020): 966-970.

S3. Deng, Jue, et al. "Electrical bioadhesive interface for bioelectronics." *Nature materials* 20.2 (2021): 229-236.

S4. Lee, Wonryung, et al. "Nonthrombogenic, stretchable, active multielectrode array for electroanatomical mapping." *Science advances* 4.10 (2018): eaau2426.

S5. Lee, Hyunjae, et al. "A graphene-based electrochemical device with thermoresponsive microneedles for diabetes monitoring and therapy." *Nature nanotechnology* 11.6 (2016): 566-572.

S6. Lee, Wonryung, et al. "Conformable microneedle pH sensors via the integration of two different siloxane polymers for mapping peripheral artery disease." *Science advances* 7.48 (2021): eabi6290.

S7. Zhao, Qinai, et al. "Highly stretchable and customizable microneedle electrode arrays for intramuscular electromyography." *Science Advances* 10.18 (2024): eadn7202.

S8. Ji, Huawei, et al. "Skin-integrated, biocompatible, and stretchable silicon microneedle electrode for long-term EMG monitoring in motion scenario." *npj Flexible Electronics* 7.1 (2023): 46.

S9. Kalevo, Laura, et al. "Effect of sweating on electrode-skin contact impedances and artifacts in EEG recordings with various screen-printed Ag/Agcl electrodes." *Ieee Access* 8 (2020): 50934-50943.

S10. Lu, Baoyang, et al. "Pure pedot: Pss hydrogels." *Nature communications* 10.1 (2019): 1043.

S11. Xu, Changshun, et al. "A PEDOT: PSS conductive hydrogel incorporated with Prussian blue nanoparticles for wearable and noninvasive monitoring of glucose." *Chemical Engineering Journal* 431 (2022): 134109.

S12. Arthur, Joshua N., et al. "PEDOT: PSS hydrogel gate electrodes for OTFT sensors." *Journal of Materials Chemistry* C 10.37 (2022): 13964-13973.

S13. Xu, Zhenying, et al. "A conducting polymer PEDOT: PSS hydrogel based wearable sensor for accurate uric acid detection in human sweat." *Sensors and Actuators B: Chemical* 348 (2021): 130674.

S14. Ajmal Mokhtar, Siti Musliha, et al. "Electrochemical stability of PEDOT for wearable on-skin application." *Journal of Applied Polymer Science* 138.44 (2021): 51314.

S15. Popov, Vasiliy I., et al. "Graphene-PEDOT: PSS humidity sensors for high sensitive, low-cost, highly-reliable, flexible, and printed electronics." *Materials* 12.21 (2019): 3477.

S16. Marozas, Vaidotas, et al. "A comparison of conductive textile-based and silver/silver chloride gel electrodes in exercise electrocardiogram recordings." *Journal of electrocardiology* 44.2 (2011): 189-194.

S17. Saadi, Hyem, and Mokhtar Attari. "Electrode-gel-skin interface characterization and modeling for surface biopotential recording: Impedance measurements and noise." *2013 2nd International Conference on Advances in Biomedical Engineering. IEEE*, 2013.

S18. Kim, Yong Min, and Hong Chul Moon. "Ionoskins: nonvolatile, highly transparent, ultrastretchable ionic sensory platforms for wearable electronics." *Advanced Functional Materials* 30.4 (2020): 1907290.

S19. Bryant, Saffron J., et al. "Non-volatile conductive gels made from deep eutectic solvents and oxidised cellulose nanofibrils." *Nanoscale advances* 3.8 (2021): 2252-2260.

S20. He, Peisheng, et al. "Moisture self-regulating ionic skins with ultra-long ambient stability for self-healing energy and sensing systems." *Nano Energy* 128 (2024): 109858.

S21. Kusama, Shinya, et al. "Transdermal electroosmotic flow generated by a porous microneedle array patch." *Nature communications* 12.1 (2021): 658.

S22. Pranti, Anmona S., et al. "PEDOT: PSS coating on gold microelectrodes with excellent stability and high charge injection capacity for chronic neural interfaces." *Sensors and Actuators B: Chemical* 275 (2018): 382-393.

S23. Somboonsub, Bongkoch, et al. "Preparation of the thermally stable conducting polymer PEDOT–Sulfonated poly (imide)." *Polymer* 51.6 (2010): 1231-1236.

S24. Kim, Minjae, et al. "Curved microneedle array-based sEMG electrode for robust long-term measurements and high selectivity." *Sensors* 15.7 (2015): 16265-16280.

S25. Krieger, Kevin J., et al. "Development and evaluation of 3D-printed dry microneedle electrodes for surface electromyography." *Advanced Materials Technologies* 5.10 (2020): 2000518.

S26. Li, Zhao, et al. "Microneedle electrode array for electrical impedance myography to characterize neurogenic myopathy." *Annals of biomedical engineering* 44.5 (2016): 1566-1575.

S27. Wang, Renxin, et al. "A microneedle electrode array on flexible substrate for long-term EEG monitoring." *Sensors and Actuators B: Chemical* 244 (2017): 750-758.

S28. Zhao, Xin, et al. "A local de-insulation method and its application in neural microneedle array." *Microsystems & Nanoengineering* 11.1 (2025): 103.

S29. Ren, Lei, et al. "Flexible microneedle array electrode using magnetorheological drawing lithography for bio-signal monitoring." *Sensors and Actuators A: Physical* 268 (2017): 38-45.

S30. Li, Junshi, et al. "Low-cost, metal-based micro-needle electrode array (M-MNEA): a three-dimensional intracortical neural interface." *2019 20th International Conference on Solid-State Sensors, Actuators and Microsystems & Eurosensors XXXIII (TRANSDUCERS & EUROSENSORS XXXIII). IEEE*, 2019.

S31. Hong, Insic, et al. "Study on the oxidation of copper nanowire network electrodes for skin mountable flexible, stretchable and wearable electronics applications." *Nanotechnology* 30.7 (2018): 074001.

S32. Kim, Jae Hun, et al. "Highly durable and flexible transparent electrode on PET based on copper and cupronickel multilayer." *Japanese Journal of Applied Physics* 62.12 (2023): 125502.

S33. Lee, Jungho, et al. "Ionic Passivation and Oxidation Dynamics for Enhanced Viability of Copper-Based On-Skin Bioelectrodes in Biological Environments." *Journal of Sensor Science and Technology* 32.6 (2023): 352-356.

S34. Ding, Su, et al. "Highly conductive and transparent copper nanowire electrodes on surface coated flexible and heat-sensitive substrates." *RSC advances* 8.4 (2018): 2109-2115.

S35. Hokazono, Masahiro, Hiroaki Anno, and Naoki Toshima. "Thermoelectric properties and thermal stability of PEDOT: PSS films on a polyimide substrate and application in flexible energy conversion devices." *Journal of Electronic Materials* 43.6 (2014): 2196-2201.

S36. Li, Junshi, et al. "High-performance flexible microneedle array as a low-impedance surface biopotential dry electrode for wearable electrophysiological recording and polysomnography." *Nano-Micro Letters* 14.1 (2022): 132.

S37. Huang, Dong, et al. "A wireless flexible wearable biopotential acquisition system utilizing parylene based microneedle array." *2019 20th International Conference on Solid-State Sensors, Actuators and Microsystems & Eurosensors XXXIII (TRANSDUCERS & EUROSENSORS XXXIII). IEEE*, 2019.

S38. Nishinaka, Yuya, et al. "Fabrication of polymer microneedle electrodes coated with nanoporous parylene." *Japanese journal of applied physics* 52.6S (2013): 06GL10.

S39. Ren, Lei, et al. "Fabrication of flexible microneedle array electrodes for wearable bio-signal recording." *Sensors* 18.4 (2018): 1191.

S40. Xiang, Zhuolin, Jingquan Liu, and Chengkuo Lee. "A flexible three-dimensional electrode mesh: An enabling technology for wireless brain–computer interface prostheses." *Microsystems & Nanoengineering* 2.1 (2016): 1-8.

S41. Shukla, Darpan, Yuxuan Liu, and Yong Zhu. "Eco-friendly screen printing of silver nanowires for flexible and stretchable electronics." *Nanoscale* 15.6 (2023): 2767-2778.

S42. Park, Jinkyung, et al. "Electromechanical cardioplasty using a wrapped elasto-conductive epicardial mesh." *Science translational medicine* 8.344 (2016): 344ra86-344ra86.

S43. Tian, Limei, et al. "Large-area MRI-compatible epidermal electronic interfaces for prosthetic control and cognitive monitoring." *Nature biomedical engineering* 3.3 (2019): 194-205.

S44. Jiang, Yuanwen, et al. "Topological supramolecular network enabled high-conductivity, stretchable organic bioelectronics." *Science* 375.6587 (2022): 1411-1417.

S45. Li, Yang, et al. "Achieving tissue-level softness on stretchable electronics through a generalizable soft interlayer design." *Nature communications* 14.1 (2023): 4488.

S46. Shi, Zhifeng, et al. "Silk-enabled conformal multifunctional bioelectronics for investigation of spatiotemporal epileptiform activities and multimodal neural encoding/decoding." *Advanced Science* 6.9 (2019): 1801617.

S47. Norton, James JS, et al. "Soft, curved electrode systems capable of integration on the auricle as a persistent brain–computer interface." *Proceedings of the National Academy of Sciences* 112.13 (2015): 3920-3925.
